# Nanoparticles-based phototherapy systems: molecular mechanisms and clinical applications

**DOI:** 10.1038/s41392-025-02536-w

**Published:** 2026-03-16

**Authors:** Deepak S. Chauhan, Rajendra Prasad, Mukesh Dhanka, Navneet Kaur, Hitasha Vithalani, Kaveesha Liyanapathirana, Roopa Hebbandi Nanjundappa, Huile Gao, Channakeshava Sokke Umeshappa

**Affiliations:** 1 Department of Microbiology and Immunology, Dalhousie University, Halifax, NS Canada; 2 Department of Pediatrics, IWK Research Center, Halifax, NS Canada; 3 School of Biochemical Engineering, Indian Institute of Technology (BHU), Varanasi, Uttar Pradesh India; 4 Department of Biological Sciences and Engineering, Indian Institute of Technology Gandhinagar, Gandhinagar, Gujarat India; 5 Department of Chemical and Biomedical Engineering, FAMU-FSU College of Engineering, Tallahassee, FL USA; 6 National High Magnetic Field Laboratory, FSU, Tallahassee, FL USA; 7 Key Laboratory of Drug-Targeting and Drug Delivery System, West China School of Pharmacy, Sichuan University, Chengdu, China

**Keywords:** Drug development, Translational research

## Abstract

Nanoparticle-based phototherapy represents a paradigm shift in precision medicine, harnessing light-activated mechanisms to modulate cellular pathways across a spectrum of diseases. By integrating nanoparticles, phototherapeutic modalities achieve enhanced light absorption and improved targeting and amplification effects, such as reactive oxygen species generation in photodynamic therapy and localized heating in photothermal therapy. Gold nanoparticles and hybrid constructs have attracted considerable attention in both photothermal and photodynamic therapies, while delivery platforms, such as liposomes and dendrimers, fine-tune biodistribution and release kinetics. At the molecular level, phototherapy induces oxidative stress, triggers apoptotic and autophagic cascades and modulates immune responses by altering cytokine profiles and T-cell activity processes, which are critical not only in cancer therapy but also in managing various chronic conditions, including cardiovascular, neurodegenerative, metabolic and autoimmune disorders. In this review, we chart the evolution of nanoparticle-based phototherapy systems by examining their core components, classification schemes and delivery platforms that drive treatment specificity. We then dissect the underlying signaling pathways, highlighting how light-triggered interventions intersect with key molecular networks in chronic disease contexts. Additionally, we critically evaluate FDA-approved agents and insights from recent clinical trials, outlining the major challenges to clinical translation, including nanoparticle optimization, efficient light delivery and regulatory hurdles. By integrating molecular insights with clinical advancements, nanoparticle-based phototherapy has emerged as a transformative, noninvasive strategy poised to revolutionize therapeutic approaches for a wide range of diseases.

## Introduction

Near-infrared (NIR) light-mediated phototherapy is a noninvasive treatment modality that uses specific light wavelengths in NIR-I (650–900 nm), NIR-1b (900–1000 nm) and NIR-II (1000–1700 nm) to treat various clinical conditions effectively, including cancer,^1^ autoimmunity,^2^ neurodegenerative diseases,^3^ skin disorders,^4^ cardiovascular diseases^5^ and even microbial infections.^6^ The importance of light-based phototherapy was inspired when sunlight was thought to have therapeutic properties during ancient civilizations.^7^ However, modern phototherapy began to take shape with the pioneering work of Niels Finsen in the late nineteenth century, who established the therapeutic potential of concentrated light for treating lupus vulgaris and was awarded the Nobel Prize in Medicine in 1903.^8,9^ Since then, phototherapy has made significant progress, moving from elementary applications to complex technologies capable of tailored therapeutic approaches. NIR light-based photodynamic therapy (PDT) and photothermal therapy (PTT) are the two main groundbreaking platforms in the field of phototherapy that have been widely reported. In PDT, photosensitizing agents produce reactive oxygen species (ROS), which cause oxidative damage to cellular constituents and ultimately result in necrosis or apoptosis.^1^ PTT is based on the light-to-heat conversion “phototransduction” of photothermal agents, which destroy targeted abnormal or diseased cells upon generating hyperthermia locally.^10^ Both therapeutic approaches have been shown to be important in preclinical and clinical settings with minimal side effects. Furthermore, these modalities provide significant benefits, including excellent spatial precision, low systemic toxicity and minimal invasiveness, making them particularly intriguing for applications in oncology.^1^ However, the clinical translation of phototherapy is hindered by several key challenges, including inadequate tissue penetration of NIR light, scalability of formulations, low bioavailability of photothermal agents and their nonspecific distribution. Despite the improved penetration of NIR-II wavelengths, biological barriers such as light scattering, absorption by endogenous molecules and tissue heterogeneity limit its reach in deep-seated tissues, which often require high-intensity irradiation that risks collateral damage. Additionally, many small molecule-based photothermal agents suffer from poor aqueous solubility, rapid systemic clearance and instability under physiological conditions, leading to suboptimal accumulation at target sites.^11^

Nanotechnology has emerged as a transformative solution to these limitations, paving the way for nanoparticle-based phototherapies that increase treatment efficacy and overcome inherent problems.^12^ Owing to their unique physicochemical features, nanoparticles (NPs) present unprecedented prospects for improved phototransduction, site selectivity, and high cargo capacity for phototherapeutic drug delivery and activation. For example, oxygen-generating NPs have been engineered to reduce tumor hypoxia, a significant barrier to effective PDT, by releasing oxygen directly into the tumor microenvironment.^13^ Nanocarriers such as liposomes^14^, dendrimers^15^, polymeric micelles^16^ and others^17^ have been studied for their ability to promote PDT, resulting in better encapsulation and controlled release of photosensitizers with improved stability and therapeutic indices. On the other hand, advanced developments in nanotechnology have led to the development of multifunctional NPs that integrate diagnostic and therapeutic properties within a single platform known as theranostics. For example, gold nanoshells, as well as magnetic NPs, can operate as both contrast agents for imaging and photothermal agents for hyperthermia therapy, allowing real-time monitoring of treatment outcomes.^18,19^ Furthermore, various NPs and their stimuli-responsive mechanisms have also been studied for use in phototherapy.^19–21^ Despite these advances, the clinical application of nanoparticle-based phototherapy remains challenging. The key challenges are (i) scalability, (ii) batch-to-batch variability, (iii) photophysical and physicochemical stability and (iv) questionable biocompatibility and biosafety, which determine regulatory approval. Furthermore, the dynamic and heterogeneous character of the tumor microenvironment needs novel approaches to improve the flexibility and robustness of these systems.^22^ For example, the use of artificial intelligence and machine learning approaches in nanoparticle design has demonstrated promise in optimizing drug delivery channels, predicting therapeutic outcomes and personalizing treatment regimens.^23^ Another essential aspect of improving nanoparticle-based phototherapy is overcoming the difficulties associated with light delivery in vivo. The limited penetration depth of the first window of NIR light (650–900 nm) in biological tissues frequently hinders the therapeutic potential of PDT and PTT, especially for deep-seated tumors.^24^ To circumvent this issue, strategies include using fiber-optic devices for endoscopic light delivery, developing implantable light-emitting systems that can provide localized illumination and altering the wavelength of NIR light to more than 900 nm.^25^ Furthermore, the development of novel photosensitizers and photothermal agents with high efficiency has increased the therapeutic response during deep-tissue phototherapy.^26^ Another area of active research is how combination therapies can improve the efficacy of nanoparticle-based phototherapy. Integrating PDT and PTT with other therapeutic modalities, such as chemotherapy, immunotherapy and/or radiotherapy, can result in synergistic effects that improve treatment outcomes.^27^ For example, NPs loaded with photosensitizers and chemotherapeutic agents can simultaneously elicit phototoxic and cytotoxic effects, effectively attacking cancer cells through multiple mechanisms.^28,29^ Similarly, phototherapy combined with immune checkpoint inhibitors has been demonstrated to stimulate robust antitumor immune responses, providing a promising approach for overcoming immune evasion by tumors.^30^

Nanoparticle-based phototherapy has shown promise in treating neurological disorders such as Alzheimer’s disease and cardiovascular conditions such as atherosclerosis.^31,32^ Functionalized NPs enable targeted hyperthermia, enzyme activation and improved drug delivery across the blood‒brain barrier (BBB). In atherosclerosis, PDT faces challenges such as photosensitivity and light delivery but has shown potential in clinical trials with various photosensitizers. Overall, NPs enhance the specificity, efficacy and safety of phototherapy.

To the best of our knowledge, nanoparticle-based phototherapy represents a paradigm shift in the treatment of cancer and other disorders, providing remarkable accuracy, adaptability and efficacy. Various nanoparticle-based phototherapy systems, with a focus on their molecular mechanisms and therapeutic implications, are discussed in the following sections. The discussion begins with a historical review, detailing the key milestones and discoveries that have sculpted the field and laid the groundwork for current advancements. Therefore, we categorized the various types of NPs employed in phototherapy and investigated their distinct functional roles and processes of interaction with light to obtain therapeutic results. The delivery systems that allow for precise targeting of diseased tissues are also examined, highlighting strategies that improve bioavailability while addressing concerns such as off-target effects. To further understand the therapeutic efficacy of nanoparticle-based phototherapy systems, we reviewed the molecular foundations, with a focus on ROS generation, apoptosis and autophagy pathways and immune modulation mechanisms that orchestrate therapeutic responses at the cellular level. Finally, we turn to the practical application of nanoparticle-based phototherapy by summarizing the progress made through FDA-approved medications and continuing clinical trials while highlighting the significant difficulties that hinder their widespread adoption. The unified narrative of this review aims to bridge the gap between molecular insights and translational applications.

### Historical overview of nanoparticle-based phototherapy systems

#### Early beginnings of phototherapy

Phototherapy has ancient origins, with its therapeutic properties well documented across various civilizations. Heliotherapy, the use of sunlight to treat ailments, was common in ancient Egyptian, Indian and Chinese medicine.^7^ Ebers Papyrus (approximately 1550 BCE) documented treatments for vitiligo via psoralen-containing extracts from plants such as *Psoralen corylifolia*, followed by exposure to sunlight.^33^ Similarly, early use of colored sheets in Chinese medicine to harness sunlight for health benefits laid the foundation for light-based therapy. Scientific advances in the 17th and 18th centuries shifted phototherapy from empirical methods to a more science-based approach. Johann Wilhelm Ritter discovered ultraviolet light in 1801, which eventually became a cornerstone of phototherapy.^34^ This period also included the birth of “sun sanatoria,” where solar radiation was utilized in therapy regimens to treat tuberculosis and other diseases.^35^ The identification of the antibacterial characteristics of light strengthened its medical potential.

Hermann Von Tappeiner, who introduced the term “photodynamic action” in the late nineteenth century, played a pivotal role in the evolution of heliotherapy (the therapeutic use of natural sunlight) into modern PDT.^33^ Photosensitizers such as eosin and Magdala red, along with artificial light, were used in this study to treat skin disorders, including basal cell carcinoma. Von Tappeiner reported that when these photosensitizers are activated by light, they produce ROS that preferentially damage diseased cells.^36^ These key findings lay the scientific groundwork for the development of PDT, although its practical application faces significant challenges. The photosensitizers available at that time had low solubility and selectivity, as well as high systemic toxicity. Nanoparticle-mediated delivery of photosensitizers enhances biodistribution, improves tumor selectivity and optimizes therapeutic efficacy, offering a more precise and effective approach to PDT.^33^

#### Introduction of nanoparticles in phototherapy

**Fig. 1:**
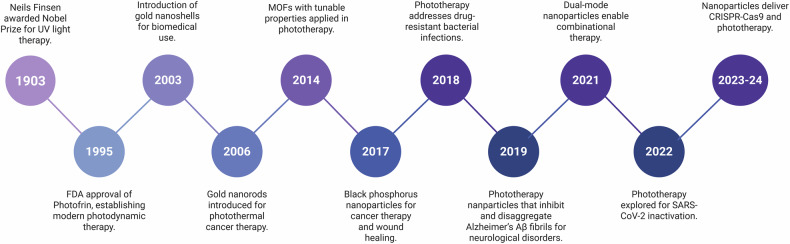
Chronological illustration of major achievements in nanoparticle-based phototherapy development. This figure highlights landmark developments from early UV light therapy to recent CRISPR‒Cas9 codelivery systems. Breakthroughs include gold nanorods, MOFs, and dual-mode nanoplatforms for combinational therapies. The graphics were created with BioRender (https://www.biorender.com)

The conceptualization of nanoparticle-based phototherapy began with the development of PDT in the 1970s,^37^ as shown in Fig. 1. Early studies investigated porphyrin-based photosensitizers, demonstrating the feasibility of PDT for cancer treatment.^33^ However, these systems have significant constraints, including limited solubility and poor tumor selectivity, prompting studies to examine NPs as delivery enhancers.

Between 1980 and 1990, gold nanoparticles (AuNPs) gained significant attention for biomedical applications owing to their distinctive optical characteristics and biocompatibility.^38^ The discovery of their plasmonic capabilities was essential, allowing PTT to emerge as a separate therapeutic technique. By the early 2000s, plasmonic NPs were integrated into a phototherapy system, with gold nanorods (GNRs) and nanoshells displaying exceptional light absorption and scattering capabilities in the NIR band.^39,40^

The 2010s witnessed the advent of multifunctional and theranostic (imaging and therapeutics by a single particle) NPs, which coupled PTT and PDT functions with imaging abilities.^41^ These hybrid systems permitted real-time monitoring and improved therapeutic accuracy, paving the way for more efficacious treatments. Advances in stimuli-responsive NPs have increased specificity by allowing regulated activation driven by triggers such as pH, temperature, or the presence of enzymes. These improvements demonstrated the versatility of nanotechnology in overcoming the constraints of traditional phototherapy.^42^

In 2014, metal‒organic frameworks (MOFs)^43^ with tunable characteristics and upconversion nanoparticles (UCNPs)^44^ gained tremendous attention by allowing precise, minimally invasive therapies with deep tissue penetration. Among them, UCNPs, which convert NIR light into higher-energy visible or ultraviolet light (required for PDT), represent a major breakthrough in this field.^20^ This ability helps UCNPs overcome the obstacle of poor tissue penetration associated with standard photosensitizers because NIR light can penetrate deeper, i.e., a few centimeters into biological tissues with minimum light scattering and absorption.^45^ Furthermore, UCNPs can be designed to selectively accumulate in solid tumors with the help of passive targeting, such as the enhanced permeability and retention (EPR) effect and/or ligand conjugation or attachment.^21^

In 2015, Spyropoulos-Antonakakis et al. explored the use of polyamidoamine dendrimers coupled with zinc phthalocyanine as a novel photodynamic treatment for atherosclerosis.^46^ The study revealed that these nanodrugs aggregated selectively on atheromatous carotid tissues, causing macrophage death and targeting atheromatous plaques. This study highlights the potential of polyamidoamine dendrimer-based devices to improve the accuracy and efficacy of PDT for cardiovascular disorders.^46^

PDT itself has emerged as a promising, minimally invasive option for managing atherosclerotic plaques. However, its broader clinical adoption has been limited by several challenges: nonspecific accumulation of photosensitizers in the skin causing photosensitivity, prolonged drug-to-light intervals ranging from 3 to 24 h after administration and difficulties in delivering light effectively to deep vascular targets.

Despite these limitations, clinical trials have explored the potential of PDT in cardiovascular applications, particularly for managing atherosclerotic plaques. However, several challenges remain, including (1) nonspecific accumulation of photosensitizers in the skin, leading to photosensitivity;^47^ (2) a prolonged drug-to-light interval, typically 3–24 h after systemic administration for most photosensitizers;^48^ and (3) difficulties in effectively delivering light to the targeted vascular structures. Nonetheless, clinical studies have demonstrated encouraging results. For example, 5-aminolevulinic acid-based PDT was investigated as an adjunct therapy after angioplasty to reduce the risk of restenosis (**NCT00187811**).^49^ Another phase I trial evaluated motexafin lutetium in patients with peripheral arterial atherosclerosis,^50^ reporting no major systemic toxicity. Other photosensitizers, including Photofrin, phthalocyanine, verteporfin and indocyanine green (ICG), have also shown potential in treating atheromatous plaques and preventing neointimal hyperplasia.^32,51^

By 2016, black phosphorus NPs had emerged as effective photothermal agents for cancer and wound treatment, overcoming limits in existing therapies because of their high efficiency and biodegradability.^52^ In 2018, nanoparticle-based phototherapy was adapted to address drug-resistant bacterial infections, indicating its flexibility in treating noncancerous conditions.^53,54^ In 2019, researchers made significant progress in treating Alzheimer’s and Parkinson’s diseases by using NPs to cross physiological barriers, increasing the potential for phototherapy applications.^55^

In 2019, Liu et al. studied GNRs for the treatment of Alzheimer’s disease, leveraging their localized surface plasmon resonance (LSPR) for NIR-driven photothermal treatment. When combined with a single-chain variable fragment and thermophilic acylpeptide hydrolase, GNRs disaggregate Aβ fibrils, reducing Aβ toxicity and enhancing enzymatic Aβ breakdown. This work demonstrated the potential of GNR-based systems as targeted, multifunctional therapies for neurodegenerative illnesses.^31^ A key advantage of this GNR-based platform lies in its ability to cross the BBB, enabling site-specific delivery of therapeutic agents directly to affected brain regions. Both in vitro and in vivo studies confirmed that this strategy enhanced neuronal survival and delayed Aβ-induced neurotoxicity, suggesting a promising and targeted solution for managing neurodegenerative diseases. Building on such innovations, the 2020 s have seen the emergence of advanced nanoparticle systems that integrate phototherapy with immunotherapy, along with the development of oxygen-independent PDT designed to address the limitations of treating hypoxic tumors. These advancements underscore the evolving role of nanotechnology in overcoming long-standing barriers in both neurological and oncological applications.

Dual-modal NPs with real-time monitoring and personalized treatment became possible in 2021, leading to the integration of diagnosis and therapy.^56^ During the COVID-19 pandemic in 2022, phototherapy was applied for SARS-CoV-2, demonstrating its potential to combat pressing global health issues.^57^ In 2023, the combination of CRISPR gene-editing tools and phototherapy represented a significant advancement in precision medicine by allowing targeted therapy for cancer and genetic diseases.^58^ In 2024, NIR circularly polarized light-responsive hybrid quantum dot (QD@L/D-Gel) hydrogels were developed, demonstrating an impressive photothermal conversion efficiency of 43% and enhanced ROS production for effective tumor phototherapy.^59^ Additionally, researchers developed DNA-tagged AuNPs designed to improve targeted photothermal therapy by optimizing nanoparticle configurations for personalized cancer treatment.^60^ In 2025, a self-assembling nanoplatform was also introduced to enhance photoimmunotherapy, significantly boosting the immune response against tumors when combined with phototherapy.^61^

Each milestone marked a significant improvement in overcoming the limits of conventional phototherapy. Early photosensitizers laid the groundwork for phototherapies, whereas the use of NPs gave clinicians unprecedented control over therapeutic precision and effectiveness. Plasmonic NPs enable more precise thermal actions, lowering systemic toxicity and increasing patient outcomes.^38^

The historical background of nanoparticle-based phototherapy systems demonstrates the astonishing transition from primitive sunlight-based treatments to sophisticated nanotechnology-driven therapies. Each milestone has contributed to overcoming obstacles in specificity, effectiveness and safety toward expanding therapeutic potential. Nanoparticle-based phototherapy shows enormous promise for revolutionizing cancer treatment and beyond, particularly with the help of continuous clinical validations and the incorporation of personalized medicine.

#### Emerging trends and future directions

The advancement of nanoparticle-based phototherapy has provided a strong framework for future innovation. In the future, several intriguing trends and conceptual breakthroughs will shape the next generation of phototherapeutic platforms.

One prominent direction is the expanded clinical translation of NIR-II phototherapy, which has received substantial attention because of its deeper tissue penetration, less autofluorescence and less light scattering than NIR-I.^62^ Since light absorption and scattering by biological tissues decrease with increasing wavelength, NIR-II-mediated phototherapies provide better imaging resolution and treatment accuracy, especially for deep-seated malignancies. Efforts are presently concentrated on the development of highly efficient NIR-II-responsive phototherapy agents with acceptable biocompatibility and photostability to allow broader translational use.^63,64^

Simultaneously, the emphasis on biodegradable and bioresorbable NPs has increased, spurred by the need to address the long-term accumulation and toxicity problems associated with inorganic nanomaterials.^65,66^ These platforms, which are made up of organic or hybrid components such polypeptides, lipids and polysaccharides, are designed to degrade completely under physiological conditions. Their intrinsic biocompatibility, along with regulated degradation kinetics, make them ideal candidates for systemic applications and repeatable clinical dosage.^67^

Another transformative notion gaining popularity is the creation of logic-gated and stimulus-responsive nanodevices, which can perform consecutive therapeutic activities in response to particular intratumoral signals, such as pH, redox state, enzyme activity, or hypoxia.^68–70^ These “smart” nanodevices use Boolean logic operations (AND, OR, NOT) to improve spatiotemporal accuracy and therapeutic selectivity, ensuring that drug release and phototherapy are activated only under defined pathological conditions. These technologies significantly reduce off-target effects and increase the therapeutic index.

Furthermore, the combination of phototherapy with immunomodulatory techniques, notably photoimmunotherapy, represents a frontier with enormous therapeutic implications.^28,71^ Photoimmunotherapy combines localized phototherapeutic damage with systemic immunological activation, promoting the recruitment of antigen-presenting cells, increasing T-cell infiltration and altering the immunosuppressive tumor microenvironment. Recent developments in nanovaccines, immunoadjuvant codelivery and checkpoint inhibitor combinations are likely to accelerate the development of synergistic photoresponsive nanomedicine regimens for cancer immunotherapy.^72^

Furthermore, artificial intelligence-guided design and predictive modeling are emerging as critical tools for improving nanoparticle formulation, biodistribution and therapeutic response.^73^ Machine learning algorithms trained on high-throughput experimental datasets can reveal crucial structure‒activity connections, providing a data-driven pathway for tailored nanomedicine creation and phototherapy protocol modification.

These converging developments in nanoparticle-based phototherapy represent a paradigm shift toward multifunctional, adaptable and patient-specific platforms. As translational issues are gradually addressed through multidisciplinary cooperation, these forward-thinking solutions show enormous potential in redefining the frontiers of nanoparticle-based phototherapy.

### Components and classifications of the nanoparticles deployed in phototherapy

**Fig. 2:**
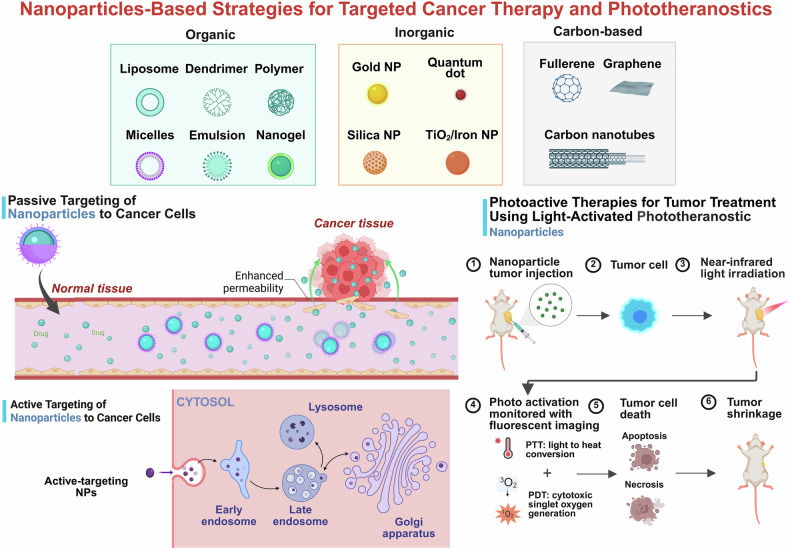
Schematic overview of different nanoparticle types and their phototherapeutic strategies: Top panels categorize nanoparticles into organic (e.g., liposomes, dendrimers, and micelles), inorganic (e.g., gold, silica, TiO₂/iron nanoparticles, and quantum dots) and carbon-based (e.g., fullerenes, graphene, and carbon nanotubes) systems. The middle-left panel depicts passive targeting via the enhanced permeability and retention (EPR) effect, allowing nanoparticles to accumulate in tumor tissues due to defective vasculature and impaired lymphatic drainage. The bottom-left panel illustrates active targeting, where nanoparticles functionalized with ligands (e.g., for G-protein-coupled receptors) selectively bind and are internalized by cancer cells. The right panels describe the mechanism of photoactive therapy: nanoparticles accumulate at the tumor site (1–2), are activated by near-infrared (NIR) light (3) and monitored via fluorescence imaging (4), leading to tumor cell death through photothermal or photodynamic effects (5) and eventual tumor shrinkage (6). The graphics were created with BioRender (https://www.biorender.com)

Nanoparticles are mainly classified into three major categories: organic, inorganic and biological.^74^ These NPs are either conjugated or self-assembled with photothermally active agents (for PTT), such as ICG, polydopamine and gold nanorods, or with photosensitizers (for PDT), such as chlorin e6 (Ce6), porphyrins and methylene blue. In addition, certain synthetic nanoparticles inherently exhibit a photothermal response upon NIR light irradiation due to their specific optical properties and intrinsic electronic (plasmonic) transitions.^75,76^ It has been reported that conjugated nanoparticles synthesized by coupling chemistry are engineered to combine organic photosensitizers with inorganic materials (such as gold, iron oxide and silica). This combination enables targeted light absorption, efficient energy transfer and improved photostability. In contrast, self-assembled hybrid nanoparticles rely on noncovalent interactions to form multifunctional structures, integrating therapeutic agents, targeting ligands and stimuli-responsive carriers in a dynamic manner. These hybrid systems optimize light-induced therapeutic mechanisms such as photothermal conversion and/or ROS generation while allowing for controlled drug release, tumor targeting and biocompatibility.^26,27^ The modular design and tunable properties of these materials make them highly promising for advancing PDT and PTT approaches in disease management (Fig. 2).

#### Photothermally active components

Photothermally active components efficiently convert absorbed light energy into heat, playing a crucial role in various applications, such as targeted therapy, tumor ablation, tissue repair/regeneration, and triggered drug delivery. Advances in nanotechnology have enabled the design of nanoscale photothermal materials with tailored optical, thermal and electronic properties.^75^ The key material categories include metallic nanostructures (e.g., Au, Pt, and Ag) with a fixed aspect ratio; semiconductors; carbon-based nanomaterials; organic polymers; and emerging materials such as MXenes, graphene and MOFs. When any chemical component of an engineered particle absorbs photons, its electrons transition to higher energy states, which exhibit phototransduction. As these excited electrons return to their ground state, the energy is dissipated as vibrational energy within the material’s lattice structure, thus producing heat. This heat generation can occur efficiently if the material minimizes radiative losses (e.g., fluorescence or phosphorescence) and maximizes nonradiative relaxation. The factors influencing this process include the material’s absorption efficiency, thermal conductivity and environmental stability under prolonged exposure to light. These materials utilize mechanisms such as plasmonic heating, nonradiative electron–hole relaxation and molecular thermal vibrations to achieve photothermal conversion, providing diverse functionalities for advanced technologies.

##### Delivery systems

**Fig. 3:**
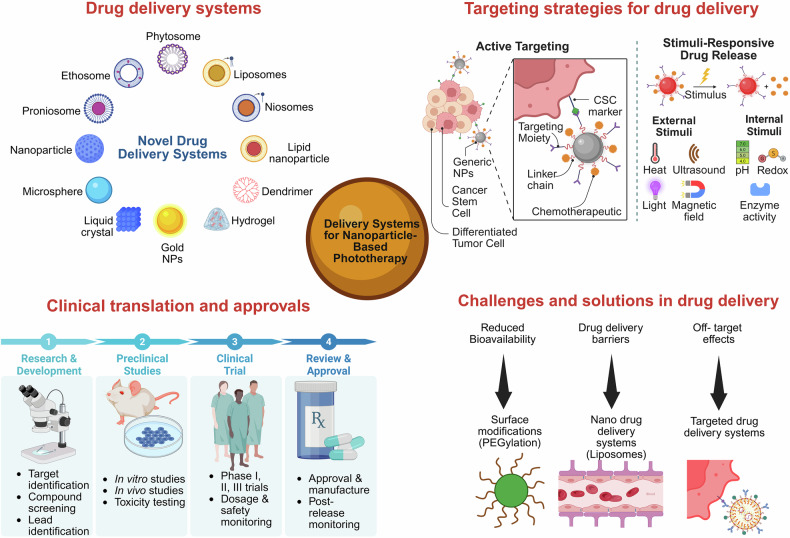
Schematic diagram of nanoparticle-based drug delivery systems for phototherapy, showing novel systems (e.g., liposomes, dendrimers, hydrogels), targeting strategies (active targeting, stimuli-responsive release), challenges (bioavailability, barriers, off-target effects), solutions (surface modifications, targeted delivery) and the clinical translation process from research to regulatory approval. The graphics were created with BioRender (https://www.biorender.com)

NPs, including organic, inorganic and surface-engineered systems, enhance phototherapy by improving targeted delivery and controlled release of therapeutic agents (Fig. 3). These platforms enable both passive and active targeting, stimuli-responsive release and codelivery of photosensitizers with chemotherapeutics, thereby integrating photodynamic, photothermal and chemotherapy for enhanced treatment efficacy.^77^

#### Optically active organic nanoparticles

Optically active organic nanoparticles have emerged as promising platforms for light-mediated localized phototherapy.^78^ Optically active NIR dyes generate thermal expansion upon light exposure, which results in sound wave signals from the localized area in the NIR region of the electromagnetic spectrum. Hence, such NIR dyes are crucial for biomedical imaging because of their ability to penetrate deeper into tissues with less absorption and scattering than visible light. NIR-responsive dyes such as ICG are widely used in medical imaging, tumor detection and intraoperative guidance owing to their FDA approval and proven safety.^79^ More importantly, these optically active dyes have been extensively researched for use in phototherapies, offering significant advantages in precision medicine and real-time visualization of solid tumor reduction. Organic nanoparticles are a class of nanomaterials composed of polymers or lipids.^80^ Owing to their unique properties, these nanoparticles are highly suitable for various applications, particularly in drug delivery and biomedical imaging. These nanoparticles are often used in phototherapy because of their biocompatibility, biodegradability and ability to encapsulate both hydrophilic and hydrophobic therapeutic agents.^80^ Furthermore, their surfaces can be modified to increase their circulation time, stability and targeting capabilities. Organic nanoparticles, such as dendrimers, hydrogels and micelles, primarily include lipid- and polymer-based systems (Fig. 3). Lipid-based delivery systems have been extensively studied for cancer therapy and other diseases, including neurodegenerative and cardiovascular conditions.^81^ **Liposomes** are microscopic spherical vesicles composed of lipid bilayers that are capable of encapsulating both hydrophilic and hydrophobic substances.^82,83^ They are widely used in drug delivery, medical treatment and pharmaceutical research because of their biocompatibility and ability to protect and transport therapeutic molecules. Their unique structure allows targeted drug delivery, reduced toxicity and improved pharmacokinetics across various medical applications.^83^ Several studies have reported the use of liposomes in phototherapy. A multifunctional liposome-based system loaded with AuNPs, graphene quantum dots and Dox and functionalized with folic acid was engineered for targeted therapy. The nanosystem demonstrated NIR-mediated tumor reduction via heat and ROS generation, offering bimodal imaging and effective cancer treatment.^84^ Moreover, these liposomes were coloaded with a photosensitive agent and an anticancer drug for synergistic therapeutic application. Liposomes have also been explored for the treatment of other diseases, including cardiovascular and neurogenerative diseases. Liposomes of different sizes were tested for myocardial accumulation and protective effects during ischemia/reperfusion in rat hearts. Compared with larger liposomes ( ~ 110 nm), smaller liposomes ( ~ 70 nm) showed 6- and 4-fold greater accumulation in the myocardium and mitochondria, respectively. Both sizes improved cardiac recovery and reduced injury, but smaller liposomes provided significantly greater protection and enhanced coronary flow and contractility, likely due to membrane-stabilizing effects.^85^ Another study explored liposomes as carriers for the sustained release of angiotensin-(1–7) in the rostral ventrolateral medulla region of the brain to assess their cardiovascular effects. Liposome-encapsulated Ang-(1–7) induced prolonged increases in blood pressure and nighttime bradycardia, significantly altering the circadian rhythms of the MAP and heart rate. These effects, lasting up to 5 days, were absent with empty liposomes, highlighting the potential of liposomes for targeted neuromodulation.^86^

**Solid lipid nanoparticles** (SLNs) offer a versatile platform for multimodal therapy, improving treatment performance while reducing drug doses and side effects. SLNs offer advantages over liposomes, such as increased stability, better protection of active ingredients and enhanced bioactivity in the spleen.^87^ They exhibit greater entrapment efficiency for hydrophobic drugs, more controlled drug delivery and enhanced flexibility in preparation, making SLNs a promising alternative for targeted therapy with improved pharmacokinetics and stability.^88^ A novel multifunctional nanosystem based on SLNs coloaded with GNRs and mitoxantrone was developed for targeted dual PTT and chemotherapy of breast cancer.^89^ These nanoparticles demonstrated suitable physicochemical properties for tumor accumulation. Under NIR irradiation (808 nm, 1.7 W cm⁻², 5 min), the system induced a temperature increase of more than 20 °C, increasing mitoxantrone release. The system was nonhemolytic and effectively targeted MCF-7 cells. Combining chemotherapy, light-induced drug release and PTT significantly increased breast cancer cell death, highlighting the potential of this lipid-based nanosystem for efficient multimodal cancer therapy. In addition to their role in cancer treatment, SLNs have also been explored for the treatment of neurodegenerative diseases. For example, a study developed solid lipid nanoparticles for delivering a capsaicin-rich extract (CPS) to counter Parkinson’s disease symptoms. The optimized SLNs showed >80% encapsulation efficiency and sustained CPS release over 24 h. They remained stable for 30 days and significantly reduced ROS levels in neuroblastoma cells exposed to oxidative stress, indicating their neuroprotective potential.^90^

**Nanostructured lipid carriers** (NLCs) are advanced colloidal drug delivery systems composed of a mixture of solid and liquid lipids, typically ranging from 50 to 500 nm in size.^91^ Compared with traditional lipid carriers, they offer improved drug loading capacity, stability and controlled release. NLCs have versatile applications in the pharmaceutical and cosmetic industries, including triggered delivery of poorly water-soluble drugs, targeted drug delivery and skin care formulations.^91,92^ Furthermore, NLCs have also been employed for cardiovascular diseases. A study developed nanostructured lipid carriers of isradipine (ISD) to increase its oral bioavailability and prolong its antihypertensive effects. The optimized formulation showed high entrapment efficiency, sustained drug release and a 4.2-fold increase in bioavailability. In vivo, ISD-NLCs protected against isoproterenol-induced myocardial damage, demonstrating both improved drug delivery and cardioprotective efficacy.^93^

**Polymeric nanoparticles**, a class of organic nanoparticles widely explored for phototherapy, are versatile structures composed of synthetic e.g., polylactic-co-glycolic acid (PLGA), polycaprolactone (PCL), polyethylene glycol (PEG) or natural (e.g., chitosan, gelatin) polymers. Their chemistry enables various formations, such as nanospheres, nanocapsules, micelles and polymersomes, each offering unique drug delivery capabilities. Examples include PLGA nanoparticles loaded with rapamycin for antiglioma activity, PCL nanoparticles with coumarin-6 for theranostics and polymer-based nanocarriers incorporating NIR-absorbing agents for photothermal therapy.^94^ A study developed hybrid polymeric nanoparticles for enhanced delivery of ICG in photothermal therapy. The nanoparticles featured a PLGA core and a pH-responsive PEG-rich coating, improving ICG photostability and reducing aggregation. Under acidic conditions, the nanoparticles released ICG more effectively, increasing its cellular uptake and anticancer efficacy in MCF-7 cells. This system demonstrated significant potential for improving ICG-mediated PTT.^95^ **Micelles** also play a significant role in phototherapy by improving the delivery and retention of therapeutic agents at the tumor site, enhancing the efficiency of light-induced therapies such as photothermal and photodynamic treatments. The ability of these materials to respond to external stimuli makes them versatile in achieving controlled drug release and localized treatment effects. For example, a study developed ICG-NH2-conjugated micelles using a PEG‒PCC copolymer to improve the tumor accumulation and therapeutic efficacy of ICG in PTT for melanoma. The micelles exhibited enhanced in vivo tumor targeting through the EPR effect, leading to complete tumor regression when irradiated with NIR light. These results suggest that ICG-conjugated micelles have great potential for both PTT and PDT, as well as imaging, in melanoma treatment.^96^

Lipid and polymeric nanoparticles are considered versatile platforms for encapsulating organic dyes as cargos, offering unique advantages in terms of biocompatibility, brightness and photothermal tunability. To achieve such photothermal agents, various strategies have been developed, including emulsion polymerization, the assembly of preformed polymers and swelling procedures, and postloading and in situ loading techniques for dye incorporation.^78,97^ Owing to the bioengineering process, their optical properties can be tuned from visible to NIR wavelengths, making them particularly valuable for in vivo imaging due to deeper tissue penetration. For example, in an optically active polymeric photothermal agent, the polymer matrices not only enhance the biocompatibility and stability of organic dyes but also reduce toxicity compared with some inorganic nanoparticles.^98^

ICG is a widely used NIR-active dye but faces challenges such as poor aqueous stability, rapid elimination and lack of specificity. To overcome these challenges, folate receptor-targeted, ICG-doped PLGA-lipid nanoparticles, which exhibit enhanced stability, targeting efficacy, prolonged circulation and potential for tumor diagnosis and imaging, have been developed.^99^ In another study, encapsulated ICG in PEG–poly(L-lysine)–poly(L-leucine) polymeric micelles exhibited a better photothermal response with a high circulation time. This system also demonstrated strong potential for localized tumor imaging and effective photothermal ablation of solid tumors, with high quantum yield and enhanced photothermal efficiency.^100^

To date, various polymeric micelles, including PEG-based,^101^ PLA-based,^102,103^ and polypeptide hybrid systems,^104^ have been explored to increase the photostability, tumor accumulation and therapeutic potential of ICG, indicating significant advancements in its applications for cancer therapy. Encapsulation in poly(styrene (styrene-alt-maleic anhydride)-block-poly(styrene)) polymeric micelles stabilizes ICG, increasing its fluorescence stability, photothermal activity and resistance to degradation. This system also shows potential for improved tumor imaging in breast cancer detection with increased stability and targeting capability.^105,106^ A novel hybrid micelle with pH and NIR light dual responsiveness was developed to overcome doxorubicin (DOX) resistance in breast cancer. It releases DOX in acidic environments, enhances tumor penetration under NIR light irradiation and effectively inhibits DOX-resistant breast cancer in vivo. The combination of lipid and polymer components improved ICG encapsulation, stability and tumor accumulation. A size-dependent strategy was developed using ICG-loaded PLGA-lecithin-PEG core-shell nanoparticles (INPs) of varying sizes to optimize drug accumulation in tumors. Among these INPs, 39-nm INPs exhibited superior cellular uptake and high photothermal efficiency, whereas 68-nm INPs achieved the best tumor accumulation and therapeutic efficacy in vivo, effectively suppressing tumor growth in a pancreatic cancer xenograft model. These findings highlight the potential of size-controlled theranostic nanoparticles for cancer imaging and therapy.^106^ Additionally, DOX- and ICG-loaded PLGA-lecithin-polyethylene glycol nanoparticles (DINPs) were developed to enhance drug delivery and photothermal efficiency. The DINPs demonstrated improved monodispersity, stability and fluorescence properties, along with increased photothermal efficiency and accelerated DOX release under laser irradiation. When combined with laser treatment, DINPs synergistically induced apoptosis in both DOX-sensitive and DOX-resistant cancer cells, significantly inhibiting tumor growth in vivo. These results underscore the therapeutic potential of DINPs for targeted cancer imaging and chemo-photothermal therapy.^107^ IR-780 iodide is also an NIR-active dye that shows superior and more stable fluorescence intensity than does ICG and has also been applied in PTT with laser irradiation. However, its lipophilicity is a major issue in therapeutic applications. application. Recently, heparin-folic acid-IR-780 nanoparticles have been studied for targeted tumor nanoparticles and have shown better circulation after being coated with polyethylene glycol.^108,109^ Compared with inorganic nanomaterials, these NIR-absorbing micelle nanoparticles offer excellent treatment efficacy with minimal safety concerns, making them promising new photothermal agents for clinical applications.^110,111^

To the best of our knowledge, optically active organic nanoparticles have also been explored for their ability to promote tissue regeneration and treat diseases such as cardiovascular and autoimmune conditions.^112–114^ Rheumatoid arthritis (RA) is a chronic autoimmune disease that causes joint damage. A novel treatment combines black phosphorus (BP) nanosheets with platelet-rich plasma-chitosan thermoresponsive hydrogels to deliver heat and ROS to inflamed joints, promote bone regeneration and protect cartilage. Hence, this approach shows promise for RA management both in vitro and in vivo in a mouse *model*. Similarly, an NIR light-triggered drug delivery system combining strontium chloride (SrCl_2_) and BP nanosheets with PLGA microspheres enhances bone regeneration. The microspheres exhibited efficient NIR absorption, photothermal effects and controlled Sr^2+^ release, promoting better cell viability, biodegradability and tissue compatibility.^115^ Another study established a multifunctional implant system for on-demand protein-based drug release tailored to individual healing needs. Using shape-memory PCL tubes activated by heat or NIR light, the system enabled precise control of protein release, as demonstrated with stromal cell-derived factor-1α. This approach has shown potential for regeneration therapies in the heart, nerves and bones.^116^

### Inorganic delivery systems

Inorganic nanoparticles offer significant advantages for phototherapy in cancer treatment because of their excellent light-to-heat conversion efficiency, particularly in materials such as AuNPs. The optical properties of these materials can be finely tuned for specific light absorption, enhancing tissue penetration, especially in the NIR region. These nanoparticles also combine PTT with drug delivery, imaging and targeting, improving therapeutic efficacy and treatment specificity.^117^ Their localized heating minimizes damage to healthy tissues, whereas surface modifications enhance tumor targeting and photothermal conversion abilities, offering a promising approach for combined tumor ablation.

**Mesoporous silica nanoparticles** are promising nanoplatforms because of their adjustable size, large surface area and high biocompatibility. MSNs with different morphologies, such as spherical and hollow structures and rods, are used for drug delivery and diagnostic imaging.^118^ Mesoporous silica-coated GNRs (Au@SiO2) were demonstrated as a multifunctional theranostic platform, combining the benefits of GNRs as both imaging and hyperthermia agents with the drug delivery capabilities of the mesoporous SiO_2_ shell. The system allows high drug entrapment efficiency and NIR-controlled drug release, enabling tailored therapy by adjusting the laser power density for chemotherapy or hyperthermia, making it a promising tool for cancer treatment.^119^

**Metallic nanoparticles:** The shape and size of AuNPs play crucial roles in determining their phototherapeutic efficacy, making them versatile tools for a range of medical applications. Among the gold nanospheres, GNRs and gold nanostars (AuNSTs) with identical mPEG-SH surface modifications, the AuNSTs presented the highest photothermal conversion efficiency for hyperthermia.^120^ Moreover, GNRs have also been studied extensively among other shaped particles. For example, GNRs coated with mesoporous silica (AuNR@mSiO2) nanoparticles loaded with DOX were explored for PTT and chemotherapy. The thickness of the mesoporous silica layers on GNRs alters the cargo capacity and therapeutic modality. For example, thin-layer ( ~ 10 nm) mesoporous silica-coated GNRs are best suited for PTT, whereas thick-layer mesoporous silica-coated GNRs are widely accepted for combined chemo-PTT applications because of their high cargo capacity. The nanoparticles, with a high photothermal conversion efficiency of 31.7%, enabled real-time drug tracking and efficient cancer treatment under femtosecond pulsed NIR laser irradiation, showing broad potential in therapeutic and imaging applications.^121^ GNRs are used for ultrasensitive in vivo spectroscopic detection and targeted PTT of head and neck carcinoma (HNC). GNRs selectively target squamous cell carcinoma (HNC) cells via an immune complex, allowing for diagnosis via spectral shift analysis and efficient NIR light absorption for PTT. This approach offers a noninvasive, nonionizing and highly sensitive diagnostic tool for micrometastasis detection while also paving the way for personalized cancer therapies.^122^ Gold nanoshells have also been explored for PTT and PDT. For example, cisplatin-loaded human serum albumin-based gold nanoshells (HCP@GNSs) were developed for synergistic chemo-PTT in lung cancer treatment. The HCP@GNSs served as both drug carriers and PTT mediators, resulting in a temperature increase upon exposure to the NIR laser that was sufficient for photothermal ablation. The combination of chemo-PTT with HCP@GNSs enhanced cytotoxicity, increased tumor necrosis and effectively cleared tumors in vivo with no adverse side effects.^123^

In addition to their role in cancer, plasmonic nanoparticles have been explored for other diseases. The effects of AuNPs and their conjugates with Simdax in a heart failure rat model were compared with those of Simdax alone, and sonoporation for enhanced delivery was evaluated.^124^ The results showed that AuNPs and the AuNPs-Simdax conjugate were biocompatible and had significant cardioprotective effects, with local delivery proving more effective than intravenous administration and sonoporation improving nanoparticle uptake by myocardial cells. Furthermore, gold-based half-shell nanoparticles functionalized with RGD peptides and loaded with methotrexate (MTX) have been developed as a targeted therapeutic strategy for rheumatoid arthritis.^125^ MTX, a widely used disease-modifying antirheumatic drug (DMARD), was encapsulated within these nanoparticles, while the RGD peptide facilitated specific targeting to inflamed joints. Upon NIR light irradiation, the Au half-shells generated localized heat, enhancing the controlled release of MTX and providing dual-mode therapy through both hyperthermia and pharmacological action. In a collagen-induced arthritis mouse model, this system achieved superior therapeutic efficacy compared with conventional MTX treatment, despite the use of only 1/930^th^ of the drug dose. The significant reduction in inflammation and joint damage highlights the potential of this NIR-responsive platform to maximize treatment outcomes while minimizing MTX-associated systemic toxicity. Moreover, this approach may be extended to other DMARDs or inflammatory disease models, offering a versatile platform for RA therapy. A bone regeneration platform was developed using inducible transgene expression and NIR-responsive hydrogels with AuNPs.^126^ NIR irradiation triggered BMP-2 release from genetically engineered mesenchymal stem cells, enhancing bone tissue formation and mineralization and demonstrating the platform’s potential for bone tissue engineering. Anisotropic gold nanobipyramids (AuNBPs) exhibit superior optical properties, making them ideal for photothermal applications.^127^ Compared with conventional sutures, AuNBP-doped laser-activated sealants embedded in a silk fibroin matrix were used for photothermal sealing of incisional wounds in mice, enabling faster skin repair and improved biomechanical properties. Gold nanoshell**s** are nanoparticles with a dielectric core coated with a thin gold shell. The optical properties of these materials can be precisely tuned by adjusting the core-to-shell ratio, making them ideal for applications in biomedical imaging, photothermal therapy and biosensing.^119^ Their ability to resonate with specific light wavelengths, particularly in the NIR region, allows for effective cancer detection and treatment, as this wavelength penetrates tissues more efficiently. Gold nanoshells are synthesized through a multistep process involving core formation, surface functionalization and gold deposition.^128^ Several studies have explored the use of gold nanoshells in cancer therapy, including cisplatin-loaded human serum albumin-based gold nanoshells (HCP@GNSs), for synergistic chemo-PTT. HCP@GNSs serve as both drug carriers for chemotherapy and efficient mediators for PTT upon NIR laser exposure, showing enhanced cytotoxicity and improved tumor clearance and immune activation without significant side effects, making them promising candidates for lung cancer treatment.^123^ Moreover, gold nanocages have been explored for cancer treatment. A study developed an in situ vaccination strategy using gold nanocages for photothermal tumor ablation combined with CpG as an immune adjuvant and JQ1 (bromodomain inhibitor) as a PD-L1 suppressor.^129^ This approach increased immune responses, activated dendritic cells, primed T cells and showed strong therapeutic effects in melanoma-bearing mice. It enhances cytotoxic T-cell infiltration, reduces PD-L1 expression and shifts macrophages from a tumor-promoting state to a tumor-fighting state, effectively remodeling the TME. Furthermore, other metal-based nanoparticles, such as platinum, palladium and porous gold, have been explored for phototherapy. While AuNPs are widely used in nanomedicine, a study showed that platinum-based multicore nanoparticles also exhibit strong NIR photothermal properties, despite the UV absorption profile of platinum.^130^ Compared with single-core nanoseeds, multicore structures demonstrated greater photothermal efficacy in glioblastoma spheroids. Researchers have confirmed the therapeutic potential of microwell arrays for high-throughput testing. X-ray absorption spectroscopy revealed that both nanoparticle types remained stable and transformed into more crystalline forms after laser exposure, highlighting their biostability and promise for cancer photothermal therapy. Porous gold nanostructures, in particular, have attracted interest because of their high surface area, tunable pore size and excellent photothermal conversion efficiency.^131,132^ The porous architecture of these materials not only allows efficient light absorption and rapid heat generation but also enables simultaneous drug loading, making them suitable candidates for combinatorial photothermal-chemotherapy applications. Additionally, the interconnected pores enhance light scattering within the nanostructure, increasing photothermal conversion under NIR irradiation. These features, along with their favorable biocompatibility and straightforward surface functionalization, position porous AuNPs as multifunctional platforms for precision oncology. Another study explored a simple, scalable method to produce palladium nanoparticles with low toxicity and strong photothermal effects.^133^ These nanoparticles were efficiently taken up by cells and selectively destroyed cancer cells under NIR light. They enabled precise cell ablation for migration studies and allowed simultaneous imaging and ablation via coherent Raman microscopy. The technique was also successfully applied for targeted tumor microablation in live mice.

**Metal‒organic frameworks** have emerged as innovative materials in phototherapy for cancer treatment and medical imaging because of their unique structural properties, including high porosity and tunable composition.^134^ These crystalline, porous hybrid materials are composed of metal ions or clusters interconnected by organic linkers, creating a unique structural framework. The high porosity of MOFs allows for efficient drug loading, and their tunable composition enables customization for enhanced therapeutic performance, including the combination of PDT, PTT and chemotherapy in a single platform. The combination of PDT and PTT, along with folic acid modification for tumor targeting, significantly inhibits tumor growth and shows strong biocompatibility, demonstrating the potential of these hybrids for effective cancer therapy.^135^

**Black phosphorus** has gained significant attention as a promising nanomaterial for phototherapy in cancer treatment and biomedical applications because of its unique physicochemical properties, including high carrier mobility, tunable bandgap and strong NIR absorption.^136^ This two-dimensional layered material consists of phosphorus atoms arranged in a puckered honeycomb structure, enabling excellent photothermal conversion efficiency and ROS generation.^137^ The high biodegradability and biocompatibility of BP further increase its potential as a theranostic agent for cancer therapy. By leveraging their tunable electronic properties, BP-based platforms can integrate PTT and PDT into a single treatment strategy, achieving precise and synergistic tumor ablation.^138^ Additionally, BP nanosheets modified with tumor-targeting ligands and responsive drug delivery systems have demonstrated superior therapeutic efficacy and minimal systemic toxicity, positioning BP as a highly versatile material for next-generation nanomedicine.^139^

### Surface engineering and modification approaches

Surface modifications and conjugation with specific targeting ligands play crucial roles in enhancing the efficacy of nanoparticles used in PTT for chronic disease treatment.^140^ These modifications serve multiple purposes, including improved tumor targeting and retention, enhanced circulation time and reduced clearance by the immune system.^141^ Various surface coating approaches, such as PEGylation,^142,143^ cell membrane camouflage,^144^ biopolymer coatings,^145,146^ and hyaluronic acid coronas,^147,148^ can be employed to achieve these goals (Fig. 3). These coatings not only provide steric stabilization and reduce opsonization but also enable active targeting through the attachment of bioactive ligands such as peptides, antibodies, or aptamers.^149^ Furthermore, multifunctional coatings can combine multiple beneficial properties, such as drug loading capabilities and additional functionalization. Modification of nanoparticle surfaces can significantly improve accumulation in diseased tissue, minimize unwanted clearance and ultimately enhance the overall efficacy of chronic disease treatment.^150,151^

In addition to surface design, understanding how nanoparticles interact with biological systems at the cellular and molecular levels is critical for optimizing therapeutic outcomes. After administration, nanoparticles are typically internalized by cells through energy-dependent endocytic pathways such as clathrin-mediated endocytosis, caveolae-mediated endocytosis and macropinocytosis.^152^ Once internalized, nanoparticles traffic through the endosomal‒lysosomal system, where acidic pH and enzymatic conditions can facilitate drug release or degradation of the nanocarrier.^153^ The surface charge, size and surface ligands significantly influence intracellular routing and endosomal escape. For example, positively charged or pH-sensitive coatings can promote endosomal membrane disruption, enhancing cytosolic drug delivery.^154^ Moreover, the intracellular fate, whether nanoparticles are recycled, degraded, or accumulate in organelles, has profound implications for phototherapy, where precise subcellular localization (e.g., mitochondria, lysosomes, or nuclei) affects the efficacy of ROS generation and hyperthermia-induced apoptosis.

#### Chemical conjugation and surface coating

Chemical conjugation modifies nanoparticle surfaces via methods such as click chemistry, thiol‒gold bonding and amide bond formation for efficient attachment. Techniques such as disulfide bonds and avidin-biotin interactions enable controlled release and targeting. The enhanced stability, selectivity and colloidal stability of functionalized AuNPs make them ideal for drug delivery, biosensing and nanoelectronics.^151^ Another study developed an assembly strategy for metallic nanostructures by click cycloaddition of GNRs and silver nanoparticles (AgNPs), creating stable, biodegradable and targetable PEG-based polymeric nanoparticles. These nanoparticles efficiently generate ultrasound signals for optoacoustic imaging and are being investigated for theranostic applications in cancer treatment, leveraging the emerging anticancer properties of AgNPs.^155^ Photothermal nanomaterials, particularly those based on polymer conjugation, offer significant potential for noninvasive cancer diagnosis and treatment. The ability of polyaniline nanoparticles to induce hyperthermia in epithelial cancer through NIR light absorption, with their optical properties tuned by protonation, was studied. Both in vitro and in vivo tests revealed that these nanoparticles effectively destroy cancer cells, highlighting their promise for cancer therapy.^156^ Polypyrrole nanoparticles conjugated with bovine serum albumin and chlorin e6 enabled both PDT and PTT, with minimal cell toxicity under dark conditions. These nanoparticles, which are labeled with Gd^3+^, offer dual-modal fluorescence and magnetic resonance imaging, showing strong tumor uptake and significantly improved therapeutic efficacy in vivo when used for combined PDT and PTT.^157^

#### Physical attachment/modification

Physical attachment, including the noncovalent interactions of nanoparticles, has been explored as a versatile approach for enhancing their functionality in PTT, allowing for the integration of therapeutic agents without altering the inherent properties of the nanoparticles.^158^ A multifunctional nanosystem that noncovalently conjugates the photosensitizer azure B to citrate-reduced AuNPs was designed, which demonstrated enhanced photothermal heating, singlet oxygen generation and DNA-targeting capabilities for trimodal anticancer therapy.^159^ To enhance antitumor efficacy, a novel nanoplatform combining porphyrin derivatives and AuNPs was developed for dual-modality PDT and PTT under laser irradiation. Chitosan-modified AuNPs were conjugated with meso-tetrakis(4-sulphonatophenyl) porphyrin (TPPS) through electrostatic interactions, resulting in TPPS/quaternized chitosan-thiol/gold hybrid NPs. These nanoparticles exhibited improved therapeutic effects, resulting in durable temperature increases ( ~ 56 °C) for PTT and efficient singlet oxygen (^1^O_2_) generation for PDT, offering a synergistic approach for the treatment of tumors.^160^ A biodegradable multifunctional nanocarrier system consisting of protein-coronated AuNPs conjugated with lonidamine (LND) and the aptamer AS1411, which combines phototherapy and chemotherapy for enhanced cancer treatment, was designed. LND was conjugated with albumin, which was then linked to AuNPs via a redox-labile disulfide bond, generating oxidative stress and ROS to kill cancer cells. The ability of AuNPs to convert photon energy into thermal heat enables synergistic photothermal/chemotherapy, demonstrating improved therapeutic efficacy and significant tumor regression in xenograft models.^161^

#### Biomimetic approaches

Cancer cell membrane (CCM)-coated nanoparticles offer a biomimetic strategy for enhancing tumor targeting and therapeutic efficiency.^162,163^ These membranes are rich in self-recognition molecules and specific adhesion proteins, which enable nanoparticles to bind selectively to homologous tumor cells, facilitating precise tumor targeting and infiltration.^164^ Unlike other cell sources, they are robust and easy to culture in vitro, making them a scalable option for obtaining biomimetic coatings. The preserved antigens and membrane structures in CCMs not only aid in immune evasion but also protect the nanoparticles from the erosive tumor microenvironment, increasing their accumulation and retention within tumors.^165,166^ Several biomimetic systems have been employed for PTT and PDT. A biomimetic drug delivery system was developed using DOX-loaded gold nanocages coated with 4T1 cancer cell membranes. This system demonstrated homotypic targeting, hyperthermia-triggered drug release under an NIR laser and effective chemo/PTT, resulting in significant inhibition of tumor growth and metastasis in breast cancer models.^167^ A NIR-responsive nanoplatform is being developed for synergistic cancer phototherapy that integrates mitochondria-targeting, PTT/PDT and oxygen-augmented PDT.

Mitochondrion-targeted delivery has gained increasing attention because of the role of organelles in regulating apoptosis and energy metabolism in cancer cells.^168^ Strategies such as conjugating nanoparticles with triphenyl phosphonium or mitochondria-targeting peptides (e.g., mitochondrial localization signals) have shown promise in enhancing mitochondrial uptake.^169,170^ These ligands exploit the negative membrane potential of mitochondria to facilitate their accumulation within the organelle. Such modifications not only improve the intracellular localization but also enhance the light-triggered responses of nanoparticles, thereby increasing their photothermal or photodynamic efficiency by inducing mitochondrial membrane disruption and initiating intrinsic apoptotic pathways. Perfluorooctyl bromide-based nanoliposomes delivered oxygen to alleviate tumor hypoxia and enhance PDT while enabling multimodal imaging for therapy guidance.^171^ While theranostic nanoparticle-based PTT shows great potential, addressing metastatic cancers remains a significant challenge. A photothermally triggered immunotherapeutic strategy was designed using magnetic-responsive immunostimulatory nanoagents (MINPs) loaded with superparamagnetic iron oxide (SPIO) nanoparticles and CpG oligodeoxynucleotides. These MINPs enable precise imaging guidance via PA/MR bimodal imaging and activate robust antitumor immune responses to target both primary and metastatic tumors, offering a promising approach for individualized cancer therapy.^172^ A biomimetic theranostic platform using ICG-loaded, cancer cell membrane-coated nanoparticles (ICNPs) was developed for cancer therapy. The ICNPs demonstrated specific targeting, immune evasion and long circulation times. With excellent photothermal response and imaging capabilities, they enable real-time monitoring and effective tumor eradication under NIR laser irradiation. The CCM shell enhanced tumor targeting and reduced organ interception, indicating that ICNPs are promising tools for cancer imaging and treatment.^173^ Chitosan-PLGA-based nanoparticles coated with CCM (MCF-7), entrapping photothermal iron oxide nanoparticles (PIO NPs), DOX and Mcl-1-siRNA were developed.^174^ The biomimetic coating enabled homotypic targeting of MCF-7/ADR cells, whereas the PIO NPs facilitated magnetic targeting, resulting in high drug uptake and enhanced accumulation at the tumor site. NIR laser irradiation and acidic pH triggered the release of DOX, increasing its cytotoxicity. The combination of chemo-PTT inhibited nearly 80% of tumor growth in an MCF-7/ADR mouse model, showing promise for the treatment of breast cancer.

### Light-mediated phototherapies and classifications

Light-mediated phototriggered therapies have gained attention for cancer treatment, bacterial therapy, tissue repair and triggered drug delivery with minimal side effects. These therapies are minimally invasive and can be combined with additional therapies such as chemotherapy, electrotherapy and immunotherapy for enhanced antitumor effects. Light-triggered therapies can be tailored to target cancer cells with various biomarkers and antibodies, making them adaptable to different types of cancers and treatment strategies. Traditional therapies rely on UV or visible light, which has limited tissue penetration. Recent advances have focused on nanosystems that can generate luminescence when excited by NIR light, ultrasound, or X-rays, offering deeper tissue penetration and enabling therapy in more challenging areas. These therapies also benefit from their ability to be precisely activated via light, which allows for on-demand drug release and tumor targeting, further enhancing their therapeutic potential. The integration of advanced technologies, such as nanoparticles and light-sensitive compounds, also increases the specificity and efficiency of treatments, reducing off-target effects and improving overall patient outcomes. There are three major types of phototherapies: PTT, PDT and photobiomodulation (PBM). However, to enhance their therapeutic effects, these therapies have been combined with immunotherapy and chemotherapy.^175^

#### Photothermal therapy

PTT uses NIR laser-absorbing agents to convert light energy into heat, resulting in cancer cell death while minimizing damage to healthy tissues.^176^ PTT offers high specificity and can be used along with other therapies, such as chemotherapy, radiation and immunotherapy, to enhance outcomes. Various photothermal nanotherapeutics, such as noble metal nanostructures and nanocarbons, have shown promising results in animal models of cancer metastasis.^177,178^ However, future studies need to address limitations such as shallow tissue penetration of light, low conversion efficiency and potential toxicity of photothermal agents. Successful PTT requires NIR light for deep tissue penetration and photothermal agents with high photothermal efficiency, good biocompatibility and tumor-specific accumulation for effective treatment and imaging.

Several nanomaterials have been explored, and among them, GNRs hold significant potential for tumor targeting and selective therapy. However, their application is hindered by the toxicity of the surfactant cetyltrimethylammonium bromide (CTAB). Replacing CTAB with polyamidoamine dendrimers and conjugating them with arginine-glycine-aspartic acid (RGD) peptides enhances the selective targeting and therapeutic effects of GNRs on cancer cells and tumors under NIR laser irradiation. In vivo studies revealed tumor disappearance in a significant portion of the test group, indicating the potential of RGD-conjugated dendrimer-modified GNRs for tumor targeting, imaging and PTT.^179^ Moreover, GNRs with strong absorption and scattering abilities in the NIR region show promise for both molecular imaging and photothermal cancer therapy. Conjugating them with anti-epidermal growth factor receptor (EGFR) antibodies allows the selective targeting of malignant cells, which can be efficiently visualized and destroyed under NIR laser irradiation, demonstrating their potential for dual diagnostic and therapeutic applications.^40^ Folic acid (FA)-conjugated nanodiamond (ND) nanoclusters were studied for selective photothermal tumor therapy.^180^ Fluorescence microscopy confirmed the selective eradication of tumors in an animal model, and compared with ND nanoclusters, FA-ND nanoclusters accumulated in tumor tissue, leading to significant tumor shrinkage after NIR laser treatment. pH-responsive PEG-decorated AuNSTs were developed that exploit extracellular pH gradients between normal and tumor tissues. PEGylated AuNSTs exhibit reversible changes in cell affinity and therapeutic efficacy, with AuNSTs modified with a nitrogen/carbon (N/C)-based functional group showing high efficacy in tumor targeting at pH 6.4 and minimal effects at pH 7.4. In vivo, AuNSTs-N/C demonstrated superior tumor accumulation and photothermal therapeutic efficacy, highlighting its potential for tumor-selective therapy.^181^ Another study developed amphiphilic redox-sensitive boron-dipyrromethene nanoparticles for PTT, imaging and drug delivery. The NPs demonstrated tumor accumulation, redox-responsive fluorescence activation and significant tumor suppression in vivo, highlighting their potential as multifunctional theranostic platforms.^182^

Several studies have explored combining magnetic targeting with PTT to enhance cancer treatment. PEGylated Fe@Fe₃O₄ nanoparticles offer three functionalities: magnetic targeting, PTT and imaging. These NPs exhibited a photothermal conversion efficiency (∼20%) comparable to that of GNRs but with superior photothermal stability. They also possess high magnetization and transverse relaxivity, making them suitable for magnetic targeting in MRI. In a xenograft HeLa tumor model, the nanoparticles effectively accumulated via magnetic targeting. This resulted in a threefold increase in MRI signal intensity and a twofold increase in temperature, resulting in efficient cancer cell ablation both in vitro and in vivo.^183^ Another study demonstrated magnetic-targeted PTT using MoS₂/Fe₃O₄ composites (MSIOs), combining MoS₂ for NIR-induced heating and Fe₃O₄ for magnetic targeting. PEG-functionalized MSIOs enable dual-modal imaging and effective, localized cancer ablation, highlighting their potential for guiding cancer theranostics.^184^

Cell membranes have been used to coat nanoparticles for PTT, enabling cell-specific targeting and controlled drug release. This study reported the use of DOX-loaded gold nanocages coated with 4T1 cancer cell membranes (CDAuNs), which selectively target homotypic tumor cells and enable hyperthermia-triggered drug release under NIR irradiation. In vivo, the CDAuNs achieved excellent chemo/PTT efficacy, significantly inhibiting tumor growth and metastasis, demonstrating their potential for breast cancer therapy.^167^ Macrophage membrane-coated gold nanoshells were developed as advanced photothermal conversion agents for in vivo cancer therapy. These biomimetic nanoparticles retained the NIR absorption of the AuNSs and demonstrated enhanced tumor targeting, prolonged circulation time and tumor accumulation. Upon NIR laser irradiation, they effectively suppressed tumor growth and selectively eliminated cancer cells, showing their potential to improve PTT outcomes.^144^ Macrophages camouflaged with DSPE-PEG-loaded NIR Ib fluorescence dye IR-792 nanoparticles (MDINPs) were developed to penetrate the BBB, enabling targeted tumor imaging and PTT. MDINPs effectively visualized orthotopic glioblastoma multiforme (GBM) and suppressed tumor growth under NIR-Ib-guided PTT, significantly prolonging survival in mice. This work offers a novel approach for integrating the diagnosis and treatment of GBM.^185^

#### Photodynamic therapy

PDT uses a photosensitizer and a specific type of light to destroy diseased cells.^186^ It involves two steps: first, the photosensitizer is administered and allowed to accumulate in cancer cells. After accumulation, the affected area is exposed to a specific wavelength of light, usually from lasers or LEDs, which activates the photosensitizer. This activation generates ROS that kill localized cancer cells, disrupt the tumor’s blood supply and stimulate the immune system to attack cancer cells in other areas.^187^ PDT is most effective for localized cancers, including skin, esophageal and lung cancers, and can also help relieve symptoms caused by obstructive tumors. The treatment is minimally invasive and typically does not damage healthy tissues or cause scarring. Since light cannot penetrate deeply into tissue, PDT is best suited for small or surface-level tumors. Potential side effects include localized pain, redness, swelling and temporary sensitivity to light. Research efforts are focused on improving PDT through advanced photosensitizers and techniques such as photoimmunotherapy, which enhances tumor targeting and enhances the immune response.^188^ PDT works by generating ROS under light and oxygen exposure, which damages cellular structures, leading to tumor cell death. Optimizing photosensitizers through structural modifications, increasing oxygen availability and integrating PDT with other therapies, such as photothermal, genetic and immunotherapy, has significantly increased their therapeutic potential.

Nanoparticle-based PDT has emerged as a promising alternative to traditional cancer treatments. NPs aid in precise tumor targeting and enhance the stability and efficiency of photosensitizers, resulting in improved treatment outcomes and reduced side effects. Researchers developed a photoresponsive nanocarrier using polyethylene glycol-block-poly(4,5-dimethoxy-2-nitrobenzylmethacrylate) (PEG-b-PNBMA) to deliver rose bengal lactone (RBL). A wirelessly activated LED implant sequentially released RBL and irradiated the tumor with light (405–580 nm), overcoming light penetration challenges and demonstrating enhanced efficacy in prostate cancer cells and 3D spheroids.^189^ Furthermore, mesoporous silica nanoparticles grafted with S-glycoside porphyrins (MSNP-PS2) have been developed, showing enhanced cellular uptake and phototoxicity, especially in UM-UC-3 bladder cancer cells, with MSNP-PS2 demonstrating the highest uptake and MSNP-PS1 exhibiting the greatest phototoxicity.^190^ One study utilized chitosan-coated liposomes to stabilize ICG and enhance its skin permeation. The chitosan coating increased the liposome size, reversed the zeta potential and protected ICG from degradation while also significantly improving the cellular uptake and photocytotoxicity in B16-F10 melanoma cells. The chitosan-coated liposomes also enhanced ICG skin permeation, highlighting their potential for effective topical PDT in melanoma treatment.^191^ Moreover, PDT is a promising treatment for urothelial carcinoma, but challenges arise from low photosensitizer solubility and poor accumulation in lesions. Au@TNA@MB nanoparticles were developed, where tannic acid-coated AuNPs were loaded with methylene blue and modified with folic acid for targeted delivery. The nanoparticles showed effective phototoxicity in cancer cells with minimal side effects in normal cells, demonstrating their potential for localized cancer treatment.^192^

Moreover, PDT has been combined with immunotherapies to improve outcomes. A key challenge is the uncontrolled generation of ROS, which can damage normal tissues and suppress immunity. Tumor metastasis also reduces its effectiveness. To address these issues, a novel nanoassembly with self-regulated photodynamic and antimetastatic properties has been developed. This system consists of chlorin e6-conjugated β-cyclodextrin, ferrocene-terminated phenylboronic acid conjugates and a rosmarinic acid-boronic acid crosslinked shell. Nanoassembly enhances stability, promotes better accumulation and regulates the generation of ROS, allowing for targeted tumor treatment while preventing metastasis and minimizing damage to healthy tissues.^193^ Another nanosystem with acid-responsive sphere-to-fiber transformation was designed to improve the distribution and accumulation of the photosensitizer chlorin e6 and immunomodulators metformin and sunitinib in tumors. This system enhances PDT efficacy, alleviates tumor immunosuppression and activates a potent immune response, significantly inhibiting tumor growth and metastasis.^194^ Furthermore, the nanosystem combines PDT with chemotherapy, using a linear triblock molecule that self-assembles into nanoparticles. In the acidic tumor microenvironment, the nanoparticles undergo charge reversal, size reduction and shape transformation into nanofibers. This process enhances tumor penetration, releases the chemotherapeutic drug berberrubine and allows the photosensitizer Ce6 to remain in the tumor longer, improving the therapeutic outcome.^195^ Moreover, a pH-sensitive supramolecular nanosystem was designed, utilizing β-CD and an acid-responsive copolymer to codeliver Ce6 and triptolide. This system demonstrated improved tumor accumulation, enhanced drug release and synergistic chemo-PDT with reduced systemic toxicity, offering a promising approach for treating tumors.^196^

#### Photobiomodulation

PBM is a noninvasive therapeutic approach that uses low-intensity light, typically in the red (600–700 nm) and near-infrared (NIR, 700–1100 nm) spectra, to modulate cellular activity and promote tissue repair.^197^ PBM works by stimulating mitochondrial cytochrome c oxidase (CCO) in the electron transport chain, leading to increased adenosine triphosphate (ATP) production, the modulation of ROS and increased nitric oxide (NO) release.^198^ These biochemical responses contribute to the regulation of cellular metabolism, the reduction of inflammation and the enhancement of tissue regeneration without causing thermal damage.

PBM has demonstrated therapeutic potential in neurology, oncology, regenerative medicine and pain management.^199^ In neurological disorders, PBM has shown efficacy in treating Alzheimer’s disease, Parkinson’s disease and stroke, as well as in improving cognitive function and neuroprotection.^200^ PBM also accelerates wound healing by promoting fibroblast proliferation, collagen synthesis and angiogenesis, making it a promising treatment for diabetic ulcers and burns.^201,202^ PBM has been shown to be effective in pain management, particularly in osteoarthritis, fibromyalgia and musculoskeletal injuries, by reducing inflammation and inhibiting pain receptor sensitivity.^203^

Despite its advantages, the PBM faces challenges in terms of dose optimization, wavelength selection and treatment standardization.^204^ The dose‒response relationship (Arndt–Schulz law) suggests that while low doses stimulate cellular activity, excessive exposure may suppress or inhibit therapeutic effects.^205^ Recent studies have focused on enhancing PBM efficacy via the use of nanoparticles, which improve light penetration, cellular uptake and targeted delivery.^202^ NP-assisted PBM is emerging as a next-generation approach for targeted and precision medicine. For example, silica-coated UCNPs and zeolitic imidazolate framework–8-coated UCNPs with NO donors were designed to convert NIR light to visible light, enhancing phototherapy in deep-tissue applications such as manipulation of neurons in the brain and spinal cord injury.^206–208^

While PBM has shown promising therapeutic applications, further research is needed to refine treatment parameters, optimize nanoparticle-assisted PBM and validate its clinical efficacy. The integration of PBM with advanced nanomaterials, imaging technologies and immunotherapies is paving the way for its clinical translation in personalized medicine.

### Targeting strategies for enhanced delivery

The success of nanoparticle-based drug delivery systems largely depends on their ability to selectively target cancer cells and tissues, making targeted delivery a key factor in their effectiveness. Various targeting strategies have been developed that can be broadly categorized into three main types: passive targeting, active targeting and active transport and retention (ATR) mechanisms (Fig. 3).^209^ Each of these approaches utilizes different biological and physicochemical properties to improve drug accumulation at tumor sites and increase the specificity of drug delivery.

#### Passive targeting

Passive targeting in nanoparticle delivery relies on the EPR effect, a characteristic of tumor physiology. Tumor blood vessels are highly permeable due to irregular endothelial gaps, thin walls and disrupted basement membranes, enabling nanoparticles to extravasate into tumor tissues. Poor lymphatic drainage further aids nanoparticle retention, facilitating their accumulation when appropriately designed for size and prolonged circulation.^210^ Features such as thin endothelial walls, disrupted basement membranes and limited pericyte coverage contribute to their leakiness. These structural abnormalities facilitate the movement of nanoparticles into tumor tissues via passive diffusion, driven by a concentration gradient. Effective passive targeting requires nanoparticles to be small enough to pass through endothelial gaps yet stable enough to circulate for prolonged periods. Size plays a crucial role, with smaller nanoparticles penetrating tumors faster, whereas larger ones are retained longer, reducing systemic clearance. For example, liposomes of approximately 90 nm effectively extravasate into tumor tissue and remain for extended durations, optimizing tumor accumulation.^211^ Unlike small molecules, the size of nanoparticles significantly impacts their blood circulation behavior, necessitating careful optimization to maximize therapeutic efficacy. NPs between 50–300 nm in size generally show slower clearance from the bloodstream, making them ideal for prolonged circulation and targeted delivery. Compared with larger Au@tiopronin nanoparticles, 50-nm Au@tiopronin nanoparticles showed superior penetration into cell monolayers, deeper spheroid penetration and greater tumor accumulation in vivo. These findings highlight the importance of smaller nanoparticles for effective tumor targeting.^212^

Current FDA-approved cancer nanotherapeutics, which rely on enhanced permeation and retention effects, provide only modest survival benefits because barriers prevent deep tumor penetration. These therapeutics, which are typically approximately 100 nm in size, accumulate around leaky tumor vasculature but struggle to penetrate dense collagen matrices. To address this, a multistage nanoparticle system in which 100-nm particles shrink to 10-nm particles after extravasation triggered by tumor-expressed proteases such as MMP-2 has been proposed.^213^ In vitro and in vivo studies using quantum dots demonstrated that size reduction enhanced diffusion and deep tumor penetration, improving drug delivery. Moreover, surface properties play crucial roles in nanoparticle internalization and drug delivery efficiency. PEGylation, the addition of polyethylene glycol, enhances nanoparticle circulation by preventing opsonization and RES clearance, thereby extending their half-life.^214^ The surface charge significantly influences nanoparticle uptake, affecting systemic circulation, tumor accumulation and intratumoral diffusion. Positive charges often enhance tumor localization by interacting with the tumor vasculature, but they limit deeper diffusion by binding with the extracellular matrix, preventing efficient tumor penetration.^215^ While positive charges can improve retention, excessive charge hinders mobility. This issue can be addressed by masking with salts or polyanionic proteins. Insights from these studies are crucial for advancing nanoparticle designs for both diagnostic and therapeutic purposes, particularly in conditions such as cancer and vascular diseases. This underscores the versatility of passive targeting in medical applications. While this approach has been used with FDA-approved liposome-based systems (e.g., Doxil^®^, Abraxane^®^), it faces limitations due to cancer heterogeneity, poor drug penetration and side effects. As a result, next-generation therapies are shifting toward active targeting, utilizing stimuli-responsive nanocarriers to improve drug delivery efficiency and reduce toxicity.

#### Active targeting

Active targeting is an advanced approach used in nanoparticle-based drug delivery systems to increase the precision and effectiveness of cancer treatments. This strategy involves functionalizing nanoparticles with specific ligands or antibodies designed to bind to receptors or antigens that are overexpressed on the surface of cancer cells.^216^ Common targets for active targeting include receptors such as human epidermal growth factor receptor 2, folate receptors and integrins, which are often upregulated in tumors to promote cell growth and survival. By attaching targeting molecules such as peptides, aptamers, small molecules, or antibodies to nanocarriers, the system can specifically bind to tumor cells, triggering receptor-mediated endocytosis and improving cellular uptake.^216^

Active targeting relies primarily on two mechanisms: ligand‒receptor binding and antibody or T-cell–receptor conjugation. T-cell receptors (TCRs) enable the precise targeting of cancer cells by recognizing specific tumor-associated antigens presented on major histocompatibility complexes (MHCs).^217^ In active targeting, TCRs can be engineered or conjugated to nanocarriers to precisely direct immune responses against cancer cells. In ligand‒receptor binding, nanocarriers are functionalized with ligands that bind to specific receptors on the cancer cell surface, such as folate receptors (FRs) or EGFR.^218^ FR targeting has been used to selectively deliver therapeutic agents to cancer cells expressing FR-α and to inflammatory cells expressing FR-β. By conjugating folate to various drug carriers, including nanoparticles, FR-targeted therapies have shown increased efficacy and reduced toxicity in preclinical studies, suggesting promising potential for clinical applications in cancer and chronic inflammatory diseases.^219^ PLGA nanoparticles encapsulating grape seed extract (GSE) were developed. By conjugating the nanoparticles with FA, the formulation specifically targeted FR-positive cancer cells, as shown through fluorescence microscopy and flow cytometry. Compared with the free drug, targeted FA-NanoGSE exhibited a threefold decrease in the IC50, enhancing bioavailability and promoting cancer cell death.^220^ Nutlin-3a (a p53 activator) and curcumin (a multidrug resistance modulator) were encapsulated in folate-functionalized PLGA nanoparticles (Fol-Nut-Cur-NPs) to increase the therapeutic efficacy of Nutlin-3a and improve treatment outcomes. The nanoparticles demonstrated increased cytotoxicity and induced apoptosis. They also modulated the expression of MDR-related genes (MRP-1, LRP) and proapoptotic and antiapoptotic proteins. This approach successfully overcomes MDR and offers a promising strategy for treating retinoblastoma by improving the bioavailability and efficacy of drugs. Transferrin (Tf) receptors constitute another type of receptor-mediated targeted delivery that offers a promising solution to increase drug circulation, decrease toxicity and increase disease specificity. The Tf receptor is upregulated in many metastatic and drug-resistant tumors, making it an ideal target for therapy, especially in gliomas, where it also facilitates crossing the BBB.^221^ For example, in gliomas, it offers a promising solution to enhance drug circulation, decrease toxicity and increase disease specificity. Another study developed a multifunctional Tf-based nanoplatform (Tf-IR780 NPs) for combined PTT/PDT with a single NIR laser. These nanoparticles demonstrated excellent tumor targeting, ROS generation and photothermal effects, achieving significant tumor suppression in vivo, highlighting their potential for targeted cancer theranostics. Antibody conjugation involves the attachment of monoclonal antibodies or antibody fragments to nanoparticles, enabling selective targeting and binding to tumor-specific antigens, thereby facilitating targeted drug delivery.^222^ These strategies improve nanoparticle accumulation at the tumor site, increase their cellular uptake and stability within target cells, increasing therapeutic efficacy and reducing side effects. Active targeting via TCR-engineered nanocarriers is an innovative approach in cancer immunotherapy that combines the specificity of T-cell receptors with the drug delivery capabilities of nanoparticles. This strategy allows for highly targeted delivery of therapeutic agents to cancer cells while minimizing off-target effects.^223^ TCRs can be engineered to recognize specific tumor-associated antigens presented by human leukocyte antigens on cancer cells. These engineered TCRs are then conjugated to nanoparticles loaded with therapeutic agents.^224^ Examples of this approach include docetaxel-loaded nanoparticles conjugated with affinity-enhanced TCRs for targeting breast cancer cells^225^ and TCR-responsive nanogels designed to deliver the IL-15 super agonist to tumor-infiltrating T cells.^223^ These advantages include increased specificity, reduced off-target effects and enhanced therapeutic efficacy. However, challenges such as human leukocyte antigen restriction, antigen selection and manufacturing complexity need to be addressed.

#### Active transport and retention

The ATR principle, proposed in 2023, provides a more accurate and clinically relevant model for nanoparticle-based drug delivery.^226^ Unlike conventional methods such as passive or active targeting, these methods offer a biologically more realistic understanding of how nanoparticles are transported and retained in tumors. NPs primarily enter tumors through energy-dependent active processes such as transcytosis rather than passive diffusion. These mechanisms involve changes in the ability of endothelial cells to transport nanoparticles across blood vessels. Once inside, nanoparticles are retained through interactions with tumor components and may exit via the lymphatic system. Drainage depends on size, with smaller vessels exiting through the peritumoral region and larger vessels exiting through the intratumoral region. The degree of retention is determined by entry, exit, and transport mechanisms and interactions within the tumor, offering insights for improving nanoparticle-based cancer therapies. This mechanism also aligns well with nanoparticle-based phototherapy strategies, where efficient tumor penetration and prolonged intratumoral retention are critical for maximizing therapeutic efficacy. By ensuring the sustained presence of photothermal or photoactivatable agents within the tumor, ATR-guided delivery can increase the precision and potency of light-triggered treatments while reducing systemic toxicity.

Studies have shown that passive transport accounts for only a small portion of tumor accumulation, with active transport being dominant, especially in human tumors lacking endothelial gaps.^227^ Active transport as the dominant entry route for nanoparticles was demonstrated in studies using 15–100-nm AuNPs in a Zombie mouse model, where passive transport accounted for only 3–25% of tumor accumulation.^228^ Electron microscopy and modeling revealed that endothelial gaps could explain only 2.5% of this uptake. The human tumor vasculature lacked such gaps, and AuNPs were observed within endothelial vesicles, indicating that transcytosis is the key pathway involved. Moreover, according to the ATR principle, the dominant route for nanoparticle exit from solid tumors is through intra- and peritumoral lymphatic vessels.^226^ Studies using AuNPs, liposomes and silica nanoparticles revealed that up to 45% of 15-nm AuNPs exited tumors within 5 days, mainly via the lymphatic system. High-resolution imaging confirmed that lymphatic vessels within tumors remain functional, with lumen sizes sufficient for nanoparticle transport. The exit route is size dependent; nanoparticles larger than 30 nm mainly exit via intratumoral lymphatics, whereas smaller nanoparticles favor peritumoral pathways. Compared with lymphatics, blood vessels contribute minimally to nanoparticle clearance from tumors.

## Molecular mechanisms in phototherapy systems

The molecular mechanisms of phototherapy systems involve a complex interplay of various systems, including ROS formation, cellular damage and programmed cell death pathways, as well as immunological regulation.^229^ These technologies leverage precision subcellular targeting to elicit specific cytotoxic effects in cancer cells while sparing healthy tissues. ROS-mediated damage triggers apoptosis and autophagy, which interact with immunological pathways to enhance therapeutic benefits.

### Reactive oxygen species generation and damage mechanisms

ROS are critical mediators of phototherapy and are produced when light-activated photosensitizers interact with molecular oxygen. Upon exposure to specific wavelengths of light, these photosensitizers transition to an excited state, enabling energy transfer to oxygen molecules through two primary photochemical mechanisms: Type I and Type II reactions.^1^

In Type I reactions, energy transfer produces radicals such as superoxide anions (^•^O_2_^−^) and hydroxyl radicals (^•^OH). These reactive species subsequently react to produce hydrogen peroxide (H_2_O_2_), which exacerbates oxidative stress.^230^ In contrast, type II reactions require direct energy transfer to molecular oxygen, resulting in singlet oxygen (^1^O_2_), a highly reactive but short-lived molecule.^231^ Singlet oxygen has a narrow diffusion radius ( ~ 155 nm), making it crucial for its precise effects. This characteristic permits the precise targeting of intracellular organelles.^232^ ROS-induced oxidative stress impairs the function of biomolecules such as lipids, proteins and nucleic acids, affecting cellular homeostasis. For example, ROS-mediated peroxidation of membrane lipids increases membrane permeability, allowing excessive calcium ion influx. This causes osmotic swelling and necrotic cell death.^233–235^

**Fig. 4:**
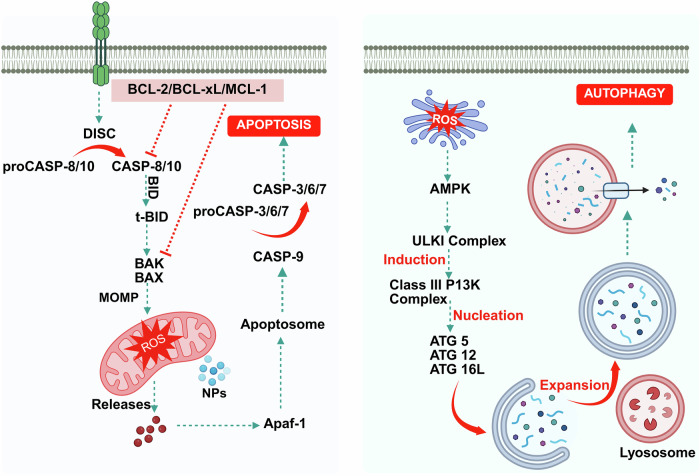
Overview of nanoparticle-mediated phototherapy-induced ROS-driven organelle damage and apoptosis. Apoptosis pathway: ROS-induced mitochondrial dysfunction triggers the release of certain proapoptotic molecules, such as cytochrome c and Apaf-1, activating the apoptotic signaling cascade and leading to programmed cell death. Autophagy pathway: Initiation by ULK1 and PI3K complexes leads to the formation of autophagosomes with LC3II and p62, followed by lysosomal fusion for the degradation of sequestered material. The graphics were created with BioRender (https://www.biorender.com). Fas Fas receptor (CD95), DISC death-inducing signaling complex, pro-CASP-8/10 Pro-Caspase-8/10, CASP-8/10 Caspase-8/10, BID BH3-interacting-domain death agonist, t-BID Truncated BID, BAK Bcl-2 antagonist killer, BAX Bcl-2 associated X protein, MOMP Mitochondrial outer membrane permeabilization, Cyt c Cytochrome c, Apaf-1 apoptotic protease activating factor-1, Apoptosome Multiprotein Complex for Caspase Activation, proCASP-3/6/7 Pro-Caspase-3/6/7, CASP-3/6/7 Caspase-3/6/7, CASP-9 Caspase-9, BCL-2/BCL-xL/MCL-1 B-cell lymphoma 2/B-cell lymphoma-extra large/myeloid cell leukemia 1, AMPK AMP-activated protein kinase, ULK1 Complex Unc-51-like autophagy activating kinase 1 complex, Class III PI3K Complex Class III phosphatidylinositol 3-kinase complex, ATG5 autophagy-related protein

As shown in Fig. 4, ROS cause severe damage to organelles, activating a variety of pathways that ultimately lead to cell death. ROS attack the mitochondrial membrane, which is required to produce ATP, the major energy source for cellular processes. This oxidative damage activates Bax, a proapoptotic protein, through the degradation of Bcl-2, an antiapoptotic protein, resulting in increased mitochondrial membrane permeability.^236^ As a result, proapoptotic molecules, such as cytochrome c, are released into the cytoplasm, which in turn activates Apaf-1.^237^ These molecules play major roles in triggering the apoptotic signaling cascade, which eventually leads to programmed cell death.^238,239^

Autophagy is a well-conserved cellular process triggered by stress signals.^240^ The ULK1 complex initiates autophagy, whereas the class III phosphoinositide 3-kinase (PI3K) complex, which contains PI3K, ATG14L, Beclin 1, VPS34 and VPS15, drives phagocytic vacuole formation. The ATG5-ATG12-ATG16 complex and LC3II promote autophagosome formation, with p62 binding to LC3II as the phagophore widens to enclose the intracellular material. Autophagosomes subsequently combine with lysosomes, where hydrolytic enzymes destroy the entrapped contents.^241–243^

In addition to the mitochondria and ER, ROS target the lysosomal membrane, leading to lysosomal membrane permeabilization. This permeabilization permits lysosomal proteases, such as cathepsins, to leak into the cytoplasm, increasing cellular damage and amplifying the destructive effects of oxidative stress.^244,245^ The selective targeting of these organelles by ROS highlights the importance of their subcellular localization in shaping the outcome of oxidative stress. In treatments such as phototherapy, the localization of photosensitizers within certain organelles is crucial for enhancing therapeutic efficacy and regulating the cellular response to oxidative stress.

### Apoptosis and autophagy pathways

The production of ROS during phototherapy triggers complex cell death pathways, notably apoptosis and autophagy. Both pathways are crucial in eliminating cancer cells and are frequently interconnected, with one pathway impacting the other depending on the intensity and duration of oxidative stress. Apoptosis, or programmed cell death, occurs via two different but interrelated mechanisms: the intrinsic (mitochondrial) pathway and the extrinsic (death receptor-mediated) pathway.^246^ The intrinsic route is driven mostly by ROS-induced mitochondrial dysfunction. Mitochondrial outer membrane permeabilization, which is caused by oxidative damage, aids in the release of cytochrome c into the cytoplasm.^247^

Cytochrome c forms a complex with apoptotic protease activating factor-1 (Apaf-1) and ATP to produce the apoptosome, which in turn activates caspase 9.^248^ This activates executioner caspases such as caspase-3 and caspase-7, resulting in the destruction of structural proteins and DNA, which causes cell death. In parallel, the extrinsic route is activated by death receptors such as Fas (CD95), whose clustering is facilitated by ROS. These receptors recruit adaptor molecules, such as Fas-associated death domain protein (FADD)^249^ and procaspase-8, to create death-inducing signaling complexes.^250^ The activation of caspase-8 in this complex either triggers the activation of caspase-3 or converts Bid into its truncated version (tBid).^251^ tBid moves to the mitochondria, boosting the intrinsic apoptotic process by increasing mitochondrial membrane permeability.^252^

In addition to causing apoptosis, ROS-induced lysosomal instability can cause leakage of cathepsins into the cytoplasm.^253^ These proteases increase mitochondrial membrane permeability by cleaving Bid and activating other proapoptotic molecules, thereby establishing crosstalk between lysosomal disruption and mitochondrial apoptosis.

Autophagy, a lysosome-dependent degradation mechanism, plays a key role in the cellular response to phototherapy.^254^ Autophagy and apoptosis are interconnected through several shared regulatory molecules and stress‒response pathways. Beclin-1, a critical initiator of autophagy, is regulated by Bcl-2 family proteins. During phototherapy, ROS can disrupt the Beclin-1–Bcl-2 complex, releasing Beclin-1 and promoting autophagy. However, under conditions of excessive stress, caspases can cleave Beclin-1, shifting the balance toward apoptosis. Furthermore, the AMPK/mTOR signaling axis regulates both autophagy and apoptosis.^255,256^ ROS activate AMPK, which inhibits mTOR and enhances autophagy under mild stress. However, chronic or excessive AMPK activation can impair mitochondrial function and trigger apoptosis.^257^ This delicate balance allows cancer cells to initially engage in autophagy as a survival mechanism, but if stress persists, the system shifts toward apoptosis, facilitating effective phototherapy-induced cell death.

ROS-induced lysosomal damage affects both autophagy and apoptosis by releasing cathepsins from permeabilized lysosomal membranes.^258^ These cathepsins activate proapoptotic molecules such as Bid, leading to mitochondrial permeabilization. This interaction between autophagy and apoptosis highlights the dynamic and context-dependent character of both processes in phototherapy. The cellular response to phototherapy is regulated by several parameters, such as the degree of damage, the type of nanoparticles utilized and the cellular environment, which determine whether autophagy is initiated or whether apoptosis is promoted. While autophagy may initially protect cancer cells from oxidative damage, prolonged stress shifts the process toward apoptosis, resulting in efficient tumor cell eradication.^259^

In addition to these traditional cell death pathways, phototherapy can cause immunogenic cell death (ICD), which is a type of apoptosis that causes immunological activation. ICD is distinguished by the exposure and release of danger-associated molecular patterns (DAMPs), including calreticulin (CRT), high mobility group box 1 (HMGB1) and ATP. These signals stimulate dendritic cells to deliver antigens and recognize tumor-associated antigens (TAAs), connecting cell death to possible anticancer immunity.

### Immune modulation and inflammatory pathways

Phototherapy systems not only induce direct cytotoxic effects but also activate the immune system, thereby increasing their therapeutic potential. ROS and singlet oxygen play critical roles in immunological activation by increasing ICD and regulating inflammatory processes.^260^

**Fig. 5:**
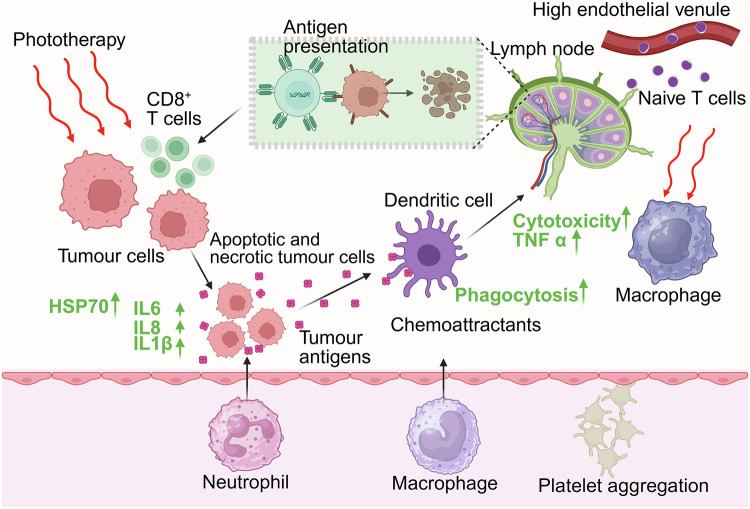
The inflammatory responses induced by phototherapy include damage to endothelial cells, resulting in arterial dilatation, platelet aggregation, cytokine production (IL-1β, IL-6, IL-8, and TNF-α) and immune cell infiltration. Necrotic and apoptotic tumor cells produce heat-shock proteins and antigens, activating dendritic cells and triggering a systemic immune response. The graphics were created with BioRender (https://www.biorender.com)

ICD is characterized by the release of DAMPs, which function as signals to the immune system.^260^ As shown in Fig. 5, ROS-induced oxidative stress causes the release of chemicals, including ATP, HMGB1 and calreticulin, from dying tumor cells.^261^ These DAMPs attract antigen-presenting cells (APCs), such as dendritic cells, and enhance the presentation of TAAs to T lymphocytes. This pathway connects the innate and adaptive immune systems, resulting in cytotoxic CD8^+^ T-cell responses to tumor cells. Calreticulin expression on the cell surface serves as an “eat me” signal for macrophages, allowing for the efficient clearance of dying cells by the immune system. Concurrently, HMGB1 and ATP act as chemoattractants and activators of APCs, enhancing T-cell priming and antitumor immunity.^262^

ROS-mediated activation of the NF-κB pathway amplifies the inflammatory response during phototherapy.^263^ NF-κB controls the synthesis of cytokines associated with inflammation, such as TNF-α, IL-1β and IL-6. These cytokines increase the recruitment of immune cells to the tumor microenvironment, hence improving the overall immune response. Furthermore, ROS stimulate the formation of hypochlorous acid (HOCl) via the peroxidase-H_2_O_2_-chloride system. HOCl acts as an immunomodulatory molecule that activates neutrophils and increases inflammation.^264^

ROS can also modify the tumor microenvironment to circumvent immune suppression. Regulatory T cells (Tregs), which decrease antitumor immunity, are downregulated in response to ROS-induced cytokine production. While ROS-mediated immune activation is an important element of phototherapy, its nonspecific nature poses a risk to healthy tissues. To mitigate these risks, localized ROS production and singlet oxygen scavengers are used. Phototherapy devices direct ROS activity to the tumor site, increasing the effectiveness of treatment while reducing side effects.^265^

Furthermore, phototherapy can reprogram tumor-associated macrophages (TAMs) from the immunosuppressive M2 phenotype (which secretes IL-10 and TGF-β) to the proinflammatory M1 phenotype (which expresses IL-12, IL-6, TNF-α and iNOS). M1 macrophages improve tumoricidal activity and attract and activate cytotoxic CD8⁺ T lymphocytes (CTLs) and CD4⁺ helper T cells, which are necessary for successful antitumor responses.^266^

Importantly, phototherapy reduces immunosuppressive cell populations such as Tregs and myeloid-derived suppressor cells, resulting in a more favorable immunological milieu for tumor elimination.^267^ Phototherapy alters the cytokine environment in tumors, increasing the levels of proinflammatory cytokines such as IFN-γ, TNF-α and IL-1β while decreasing the levels of immunosuppressive cytokines such as IL-10 and TGF-β. These changes not only increase rapid immune activation but also help develop long-term immunological memory, potentially preventing tumor recurrence.^268,269^

## Phototherapy-driven modulation of pathways in chronic disease treatment

Phototherapy, including nanoparticle-assisted strategies, leverages light-induced effects such as ROS generation and heat production to modulate cellular pathways. These effects modulate oxidative stress, inflammation, apoptosis and immune regulation. By targeting pathological processes at the molecular level, phototherapy has emerged as a promising minimally invasive approach for treating chronic diseases such as cancer, neurodegenerative disorders, cardiovascular conditions, and autoimmune diseases.

### Cancer

**Fig. 6:**
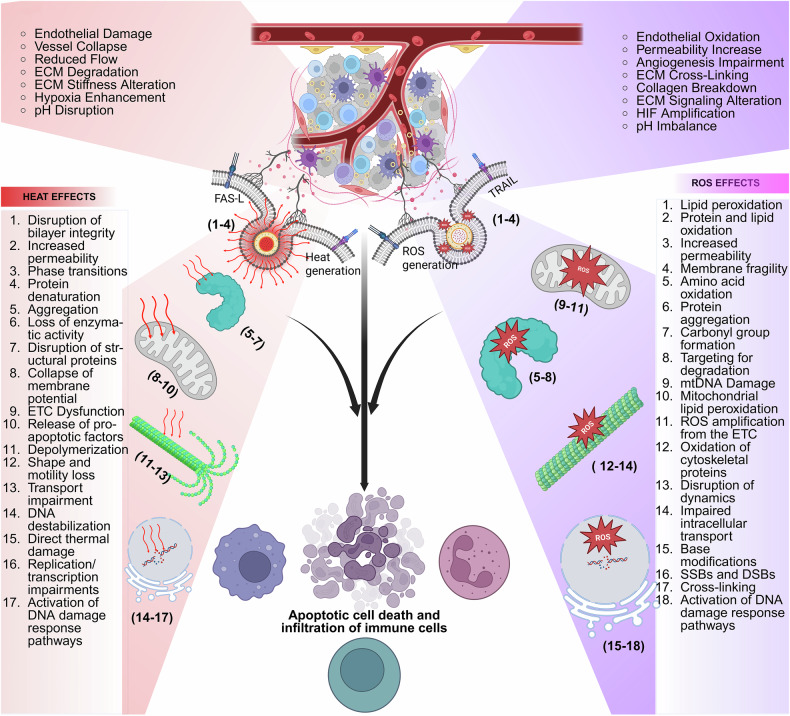
Effects of phototherapy on cellular structures and the tumor microenvironment. The left panel (red) highlights heat-induced cellular and molecular damage. The right panel (purple) highlights ROS-induced damage. The top section highlights damage to the tumor microenvironment. The combined effects of heat and ROS lead to apoptotic cell death and immune cell infiltration, as shown in the central panel, thereby promoting therapeutic efficacy in cancer treatments. The graphics were created with BioRender (https://www.biorender.com). ECM extracellular matrix, HIF hypoxia-inducible factor, ETC electron transport chain, HSP heat shock protein, SSBs and DSBs single-strand breaks and double-strand breaks

Phototherapy has emerged as a promising approach in cancer treatment because of its ability to specifically target malignant cells while sparing normal tissues.^270^ Techniques such as PDT, PTT, the combination of PDT and PTT and photoimmunotherapy can aid in light-induced reactions to achieve therapeutic effects (Fig. 6 & Tables 1, 2).

**Table 1:** Overview of different types of delivery systems for the treatment of chronic diseases

Type of nanoparticle	Major type	Type of delivery system	Key features	Applications	Ref
NFGL	Lipid-based	Liposome	Multifunctional, folic acid functionalized	NIR-mediated tumor reduction, bimodal imaging	^84^
GNOLs		Liposome	pH-responsive, controlled drug release	Chemo-PTT	^527^
GNPs&DOX-TLs HGNPs&DOX-TLs		Liposome	Thermosensitive, NIR-responsive	Synergistic chemo-PTT	^528^
ID@TSL-Gd NPs		Liposome	Coencapsulation of ICG, DOX and Gd chelates	Triple-modal imaging, chemo-, photothermal and PDT	^529^
SLNs with GNRs and mitoxantrone		Solid Lipid Nanoparticle	Folic acid functionalized	Targeted PTT and chemotherapy	^89^
P18 N PI ME-loaded SLNs		Solid Lipid Nanoparticle	Smallest SLN formulation exhibiting the highest photodynamic activity	Enhanced PDT effects, selective phototoxicity	^530^
GNP-incorporated SLNs		Solid Lipid Nanoparticle	Photothermally triggerable	Photothermal release of payloads	^531^
5-ALA loaded NLCs		Nanostructured Lipid Carrier	Controlled drug release, improved skin permeation	Topical PDT	^532^
NLC10q		Nanostructured Lipid Carrier	High encapsulation efficiency (96%), stable	PDT	^533^
PLGA-PEG hybrid NPs	Polymer-based	Polymeric Nanoparticle	pH-responsive, improved ICG photostability	Enhanced PTT	^95^
PEG-PLGA NPs with Croc dye		Polymeric Nanoparticle	pH-sensitive, iRGD conjugated	Multiplexed photoacoustic imaging, pH-responsive PTT	^534^
Por-DPP ONPs		Polymeric Nanoparticle	Multiplexed photoacoustic imaging and enhanced photothermal cancer therapy	Photoacoustic imaging-guided PTT	^534^
L1057 NPs		Semiconducting-Polymer Nanoparticle	High NIR-II brightness, stability, biocompatibility	NIR-II imaging, PTT	^535^
PHG		Hydrogel	Self-healing, Photoconversion Efficiency ~41%	Chemo-PTT	^536^
DHP@BPP		Peptide-based	Light-triggered	chemo-photodynamic-immunotherapy.	^195^
SUN-NPA		Peptide-based	immunomodulators, metformin and sunitinib	immunosuppressive effects against primary tumor and circulating tumor cells	^194^
GdCFS		Peptide-based	GSH-responsive and shape transformable nanotheranostics	MRI-guided enhanced PDT	^537^
Ce6-CD/Fc-pep-PEG		Peptide-based	Self-delivered supramolecular nanomedicine	PDT of Breast Cancer and Bone Metastases	^538^
MA-pepA-Ce6 NP		Peptide-based	Metformin mediated PD-L1 downregulation in combination with PDT	Photodynamic-Immunotherapy for Treatment of Breast Cancer	^539^
TPL + Ce6 NPs		polymer micelles	pH-sensitive supramolecular nanosystem	chemo-PDT	^196^

*NFGL* liposome-based nanotheranostics loaded with AuNPs and emissive graphene quantum dots, *GNOLs* gold nanoshell-coated oleanolic acid liposomes, *GNPs&DOX-TLs and HGNPs&DOX-TLs* doxorubicin-loaded thermosensitive liposomes, *ID@TSL-Gd NPs* idarubicin encapsulated in thermosensitive liposomes conjugated with gadolinium nanoparticles, *SLNs@GNRs–mitoxantrone* solid lipid nanoparticles incorporating gold nanorods and mitoxantrone, *P18 N PI ME-loaded SLNs* solid lipid nanoparticles loaded with purpurin-18-N-propylimide methyl ester, *GNP@SLNs* solid lipid nanoparticles incorporating AuNPs, *5-ALA-loaded NLCs* nanostructured lipid carriers loaded with 5-aminolevulinic acid, *PLGA-PEG hybrid NPs* hybrid nanoparticles composed of PLGA and PEG, *PEG-PLGA NPs with Croc dye* PEG-PLGA nanoparticles conjugated with croconaine dye, *Por-DPP ONPs* porphyrin-diketopyrrolopyrrole organic nanoparticles, *L1057 NPs* theranostic system based on semiconducting polymer nanoparticles, *PHG* plasmonic hydrogel, *SUN-NPA* chlorin, metformin and

**Table 2:** Detailed overview of the key molecular and cellular pathways targeted by nanoparticle-based phototherapy across various disease categories

Disease category	Mechanism	Targeted pathway and therapeutic effects	Ref.
Cancer	Enhances tumor ablation and immunotherapy by localizing heat and ROS, integrating photothermal and photodynamic therapy.	Induces apoptosis, DNA damage and heat shock proteins; Modulates cytokines for tumor suppression	^540^
Cardiovascular Diseases	Targets ROS and heat for plaque reduction and vascular repair, complementing other treatments.	Regulates cytokines, oxidative stress and endothelial function; Stabilizes plaques via apoptosis modulation.	^342^
Neurodegenerative Diseases	Reduces β-amyloid accumulation and oxidative stress, promoting neuroprotection.	Enhances β-amyloid clearance, ROS-mediated repair and heat shock protein activation; Lowers neuroinflammation	^541,542^
Autoimmune Diseases	Modulates immune responses via localized heat and ROS, improving immunotherapy efficacy.	Suppresses pro-inflammatory cytokines, regulates T cells and promotes immune homeostasis.	^423,543^

#### Role of reactive oxygen species and hyperthermia

ROS and hyperthermia play central roles in the therapeutic effects of PDT and PTT, acting as potent agents for oxidative and thermal damage in cancer cells. In PDT, light activation of a photosensitizer triggers energy transfer processes that generate ROS, including singlet oxygen and free radicals. This induces oxidative stress in cancer cells, resulting in selective cytotoxicity.^271^ Similarly, PTT leverages photothermal agents to convert absorbed light into heat, inducing hyperthermia that disrupts cellular structures and enhances ROS-mediated damage in combination with PDT. The preferential accumulation of photosensitizers and photothermal agents in tumor tissues ensures minimal damage to surrounding healthy cells. Given their complementary mechanisms, in which PDT relies on ROS generation and PTT relies on heat-induced stress, the combination of PDT and PTT has emerged as a rational strategy to increase antitumor efficacy, overcome tumor heterogeneity and reduce treatment resistance. This synergy not only amplifies cell death via multiple pathways but also addresses the limitations of each therapy when used alone. Therefore, the integration of both modalities via nanoparticle platforms has gained increasing attention in precision oncology. The factors influencing ROS generation and hyperthermia include the light wavelength, photosensitizer or photothermal agent type and oxygen availability in the tumor microenvironment.^271,272^

ROS and hyperthermia cause damage to critical cellular components, including lipids, proteins and nucleic acids.^273^ Lipid peroxidation compromises the integrity of cellular membranes, leading to increased membrane permeability and eventual cell lysis. Protein oxidation disrupts enzymatic activity, alters cellular signaling pathways, interferes with structural proteins and results in impaired cellular function.^274,275^ The DNA damage caused by ROS includes strand breaks and base pair modifications, which can trigger cell cycle arrest and apoptosis.^276^ Additionally, hyperthermia from PTT exacerbates cellular stress, denatures proteins, induces heat shock responses, damages DNA and amplifies ROS-induced cytotoxicity when combined with PDT. These combined effects contribute to the selective eradication of cancer cells.

The efficiency of ROS- and hyperthermia-mediated cancer cell destruction depends heavily on the photosensitizer or photothermal agent used and the light wavelength applied.^272,277^ Common photosensitizers include porphyrins, phthalocyanines and chlorins, each of which are activated by specific light wavelengths, typically in the range of 600–800 nm.^278^ This range, known as the therapeutic window (transparent to most biological fluids and tissues), allows deeper tissue penetration, making it particularly effective for treating solid tumors. Similarly, GNRs and other nanoparticle-based photothermal agents absorb NIR light, generating localized heat and complementing ROS-induced damage if combined with PDT. Moreover, advancements in nanoparticle-based photosensitizers and photothermal agents, such as upconversion nanoparticles and GNRs, have enhanced ROS generation, photothermal conversion efficiency and tumor-targeting capabilities, improving therapeutic outcomes.^279,280^ These nanoparticles often exhibit enhanced photostability, efficient ROS generation and active tumor-targeting properties, making them ideal for precision oncology applications.

#### Induction of apoptosis

Phototherapy-induced apoptosis is a key mechanism for eliminating cancer cells and involves both intrinsic and extrinsic pathways. The intrinsic (mitochondrial) pathway is activated when phototherapy generates ROS or heat stress, leading to mitochondrial membrane permeabilization. This results in the release of cytochrome c, which forms an apoptosome complex and activates caspase-9, ultimately triggering downstream caspases such as caspase-3.^281,282^ The extrinsic pathway is initiated by phototherapy-induced upregulation of death receptors, such as Fas or TRAIL receptors, on the cancer cell surface.^283,284^ Ligand binding to these receptors activates caspase-8, which subsequently activates effector caspases.

PTT generates localized hyperthermia through the absorption of NIR light by nanoparticles such as GNRs, nanoshells, carbon nanotubes and others, which can increase apoptosis by increasing caspase activation.^39,162,285,286^ Heat stress sensitizes cancer cells to apoptotic signaling by disrupting protein folding, impairing heat-shock protein activity and further destabilizing cellular homeostasis.^287^ This dual effect of phototherapy, which combines ROS generation and hyperthermia, enhances therapeutic efficiency against resistant cancer cell populations.^288^

Notably, nanoparticle-mediated phototherapy has shown promise in overcoming the limitations associated with traditional therapies.^289,290^ Functionalized nanoparticles, such as liposomes or polymeric nanoparticles encapsulating photosensitizers and conjugated with targeting ligands, enable precise delivery to cancer cells while minimizing off-target effects. Additionally, dual-modality systems combining PDT and PTT within a single nanoparticle platform have demonstrated superior therapeutic outcomes by simultaneously inducing oxidative stress and hyperthermia-induced apoptosis.^291^ For example, gold and copper-based nanoparticles are widely studied for their combined photothermal and photodynamic properties, offering a multifaceted approach to enhance tumor eradication.^292,293^

#### Immune modulation

In addition to direct cytotoxic effects, phototherapy can also modulate the immune system to promote antitumor responses, making it a powerful tool for long-term cancer control. It induces ICD by releasing DAMPs, such as calreticulin, HMGB1 and ATP, from dying cancer cells. These DAMPs act as “danger signals” that activate dendritic cells and promote antigen presentation, leading to robust activation of cytotoxic T lymphocytes.^294^ This immune activation can result in systemic antitumor effects and prevent cancer recurrence.^294^

As described earlier (see section 4.3), phototherapy reprograms the tumor microenvironment by inducing a shift in tumor-associated macrophages from the M2 phenotype to the M1 phenotype and increasing proinflammatory cytokine production.^268,295^ This immunomodulation promotes T-cell infiltration, activation and cytotoxicity, contributing to a robust and coordinated antitumor immune response.^296–299^

NPs play a critical role in enhancing the immune modulatory effects of phototherapy. For example, gold nanorods conjugated with CpG oligonucleotides have been shown to induce ICD and promote dendritic cell maturation.^300^ Hybrid nanocarriers, such as PLGA-based systems coloaded with photosensitizers and TLR agonists, have also demonstrated enhanced antigen presentation and CTL activation.^301^ Nanoparticles designed to codeliver photosensitizers and immune adjuvants, such as CpG oligonucleotides, to induce ICD and activate dendritic cells amplify antitumour immunity.^302,303^ Additionally, photoresponsive nanoparticles that release immune-stimulating agents upon irradiation offer precise control over immune activation, reducing the risk of systemic inflammation.^304^ Another notable example includes black phosphorus-based quantum dots functionalized with anti-PD-L1, which enable synergistic photoimmunotherapy by combining checkpoint blockade and hyperthermia.^305^ Another innovative approach involves the use of biomimetic nanoparticles coated with the membrane of macrophages, which enables efficient tumor antigen presentation and potent immune activation.^162^

The integration of phototherapy with immune checkpoint inhibitor therapies, such as anti-PD-1 or anti-CTLA-4 antibodies, represents a promising strategy for enhancing treatment outcomes.^71^ By combining the local tumor destruction and systemic immune activation of phototherapy with the ability of checkpoint inhibitors to overcome immune suppression, this approach has demonstrated synergistic effects in preclinical models and early-phase clinical trials.^71,306^ This combination strategy has the potential to address tumor heterogeneity and improve patient survival rates.

Among the various signaling pathways affected by phototherapy in cancer, the PI3K/Akt/mTOR and MAPK pathways both govern cell survival and proliferation but respond differently to ROS and thermal stress.^307–309^ PI3K/Akt signaling is often downregulated following oxidative insult, leading to apoptosis, whereas MAPK activation can result in either apoptosis or survival, depending on the ROS dose and exposure duration. Similarly, pathways such as the JAK/STAT and NF-κB pathways are modulated in opposite directions depending on phototherapy parameters, indicating that pathway crosstalk is highly context- and dose dependent.^310,311^ These dynamic interactions suggest that rational design of phototherapeutic protocols must account not only for target selection but also for pathway interplay and temporal activation patterns.

In summary, phototherapy leverages hyperthermia, ROS generation, apoptosis induction and immune modulation to achieve therapeutic effects in cancer treatment. The incorporation of advanced nanoparticle technologies and combination strategies further enhances their efficacy, paving the way for more effective and personalized cancer therapies. Continued research into the optimization of light delivery systems, the development of novel photosensitizers and the exploration of combination therapies will likely expand the clinical utility of phototherapy in oncology.

### Cardiovascular diseases

**Fig. 7:**
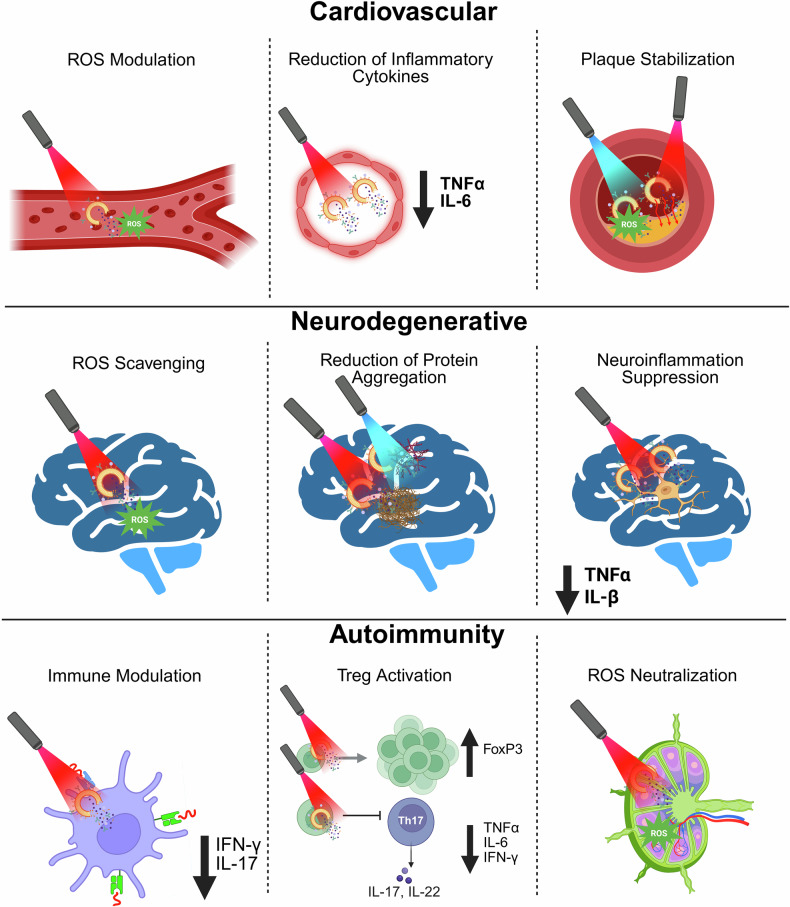
Effects of phototherapy on cardiovascular, neurodegenerative and autoimmune diseases. Top row: Phototherapy reduces oxidative stress, decreases TNFα and IL-6 levels, stabilizes plaques and improves vascular function. Middle Row: ROS scavenging and heat reduce protein aggregation and neuroinflammation, protecting neurons from oxidative and inflammatory damage. Bottom row: Phototherapy modulates immune responses by suppressing IFN-γ and IL-17, activating FoxP3+ Tregs to reduce inflammation and neutralizing ROS to restore immune balance. The graphics were created with BioRender (https://www.biorender.com)

Phototherapy has been increasingly explored for its therapeutic potential in treating cardiovascular diseases. By targeting key cellular and molecular pathways, phototherapy contributes to endothelial repair, a reduction in oxidative stress and the modulation of inflammatory responses, ultimately improving vascular and cardiac health (Fig. 7 & Table 2). Unlike cancer phototherapy, where ROS generation is used to induce cytotoxicity, phototherapy in cardiovascular applications seeks to reduce oxidative stress by scavenging ROS and enhancing endogenous antioxidant activity.

#### Effects on endothelial cells

Endothelial dysfunction is a hallmark of cardiovascular diseases. Phototherapy can restore endothelial function and promote vascular repair through precise mechanisms. Phototherapy, which uses red (620–700 nm) and NIR (700–850 nm) light, enhances endothelial cell proliferation and migration by stimulating mitochondrial activity via cytochrome c oxidase activation.^312,313^ This process increases ATP production, driving angiogenesis and vascular repair in ischemic tissues. NPs improve the efficacy of light absorption and delivery to target tissues, ensuring localized effects.^314,315^ Such advancements enable the regeneration of damaged blood vessels, improving the oxygen and nutrient supply to compromised regions. Moreover, phototherapy facilitates the upregulation of vascular endothelial growth factor (VEGF), a critical mediator of angiogenesis.^316^ VEGF activation enhances endothelial cell survival and proliferation, promoting the formation of new capillaries in ischemic areas. Studies have shown that combining phototherapy with VEGF-loaded nanoparticles amplifies these effects, resulting in accelerated recovery of blood flow and tissue perfusion.^312,313,316,317^

Abnormal vascular smooth muscle cell (VSMC) proliferation and migration contribute to pathological vascular remodeling in conditions such as atherosclerosis and restenosis.^318–320^ Phototherapy suppresses these processes by modulating signaling pathways, including the PI3K/Akt and MAPK pathways, and by supporting physiological repair mechanisms.^321^ For example, UCNPs doped with rare-earth elements enable targeted phototherapy in deeper vascular tissues, controlling VSMC behavior and preventing adverse vascular remodeling.^322–324^ Additionally, phototherapy-mediated inhibition of matrix metalloproteinases reduces extracellular matrix degradation, preserving vascular integrity and preventing plaque destabilization.^325^

#### Reduction in oxidative stress

Oxidative stress is a key contributor to cardiovascular diseases, and the ability of phototherapy to mitigate ROS levels plays a critical role in improving cardiovascular outcomes. Phototherapy, particularly in the NIR range (700–850 nm), stabilizes mitochondrial function, reducing the production of ROS and preventing oxidative damage to lipids, proteins and DNAs.^326,327^ Gold and titanium dioxide nanoparticles act as effective photosensitizers, enhancing light absorption and the ROS scavenging process. This process mitigates oxidative damage in cardiovascular tissues, preserving vascular structure and reducing the progression of diseases such as atherosclerosis.^326,327^ In addition to reducing ROS production, phototherapy stimulates the activity of endogenous antioxidants such as superoxide dismutase (SOD) and catalase, which neutralize ROS and reduce lipid peroxidation.^328^ Nanoparticle-based antioxidant strategies have shown significant promise in enhancing the ROS-scavenging effects of phototherapy. For example, cerium oxide nanoparticles exhibit regenerative antioxidant activity because of their ability to shift between Ce³⁺ and Ce⁴⁺ states, mimicking SOD and catalase enzymatic functions.^329^ Similarly, manganese dioxide (MnO₂)-based nanostructures can decompose hydrogen peroxide into oxygen, alleviating hypoxia and oxidative stress in vascular tissues.^330^ Moreover, mesoporous silica nanoparticles functionalized with antioxidant molecules such as quercetin allow for sustained and targeted ROS neutralization, improving endothelial protection.^331^

The use of nanocarriers with antioxidant properties enhances the therapeutic efficacy of phototherapy by ensuring sustained delivery to vascular tissues.^332,333^ This dual approach prevents the formation of toxic byproducts such as malondialdehyde (MDA), which contribute to endothelial dysfunction and vascular damage.^332–334^

Furthermore, phototherapy enhances NO bioavailability, a critical factor in maintaining vascular health.^335–337^ By modulating endothelial nitric oxide synthase activity, phototherapy promotes NO production, leading to improved vasodilation and reduced oxidative stress. Nanoparticles conjugated with NO donors or photoresponsive NO-releasing agents have been developed to amplify this effect, offering a promising strategy for treating hypertension and other vascular disorders.^338^ NO photodonors release NO upon light activation, enabling precise vascular-targeted phototherapy. In cancer and cardiovascular treatments, they enhance vasodilation and drug delivery, with NIR-triggered nanoparticles improving tissue penetration. Recent studies, such as those using upconversion nanoparticles with NO precursors, have shown promise in targeting the tumor vasculature, although clinical translation needs further safety validation.^339^

#### Inflammatory pathways

Chronic inflammation underlies many cardiovascular diseases, and phototherapy has demonstrated the ability to modulate inflammatory pathways and reduce disease progression. Phototherapy, which uses red (600–700 nm) and NIR (700–1100 nm) light, suppresses proinflammatory cytokines such as TNFα and IL-6, which drive vascular inflammation and endothelial dysfunction.^340^ NPs conjugated with anti-inflammatory agents have been shown to increase the precision of phototherapy, ensuring targeted suppression of cytokines in inflamed tissues.^341,342^ This effect slows the progression of atherosclerosis and reduces ischemic damage in cardiac tissues.^340,341^

NF-κB is a key transcription factor that regulates the expression of inflammatory mediators. Phototherapy inhibits NF-κB activation in endothelial cells, macrophages and VSMCs, thereby attenuating inflammatory responses.^343^ Nanoparticles such as graphene and MXene-based nanocarriers combined with light therapy improve penetration and efficiency, reducing inflammation at the molecular level.^343–345^ These approaches limit plaque formation in atherosclerosis and minimize myocardial damage following ischemia. In addition to targeting proinflammatory pathways, phototherapy enhances the production of anti-inflammatory cytokines, such as IL-10, which counteract the effects of chronic inflammation.^346^ By shifting the balance toward an anti-inflammatory phenotype, phototherapy contributes to the resolution of vascular inflammation while promoting tissue healing. Photoresponsive hydrogels for immunomodulation have been developed to achieve localized delivery, further improving therapeutic outcomes.^347,348^

Phototherapy ( < 48 °C) also influences macrophage polarization, shifting them from a proinflammatory M1 phenotype to an anti-inflammatory M2 phenotype.^349,350^ This shift reduces the production of inflammatory mediators and promotes tissue repair. The integration of phototherapy with nanoparticles enables the targeted delivery of polarization-inducing agents, such as microRNA mimics or inhibitors, to enhance therapeutic effects or remove inflammatory macrophages, creating a synergistic approach to managing cardiovascular inflammation.^351–353^

Emerging evidence suggests that phototherapy can modulate the gut microbiome, a key player in systemic inflammation and cardiovascular health.^354^ By targeting gut dysbiosis (i.e., imbalances or alterations in the composition of the gut microbiota), phototherapy may indirectly reduce the levels of circulating inflammatory markers and improve vascular function.^355^ This novel area of research highlights the potential for integrative approaches that combine phototherapy with dietary or probiotic interventions to address cardiovascular diseases.

In cardiovascular diseases, phototherapy modulates several interconnected signaling pathways that affect endothelial function.^356^ For example, the PI3K/Akt/eNOS and AMPK/SIRT1 pathways both promote NO production, which is crucial for vasodilation and endothelial repair.^357^ However, while PI3K/Akt is more sensitive to oxidative stress and regulates acute NO synthesis, AMPK activation also enhances mitochondrial biogenesis and long-term metabolic support in endothelial cells.^358,359^ Conversely, NF-κB signaling, which is often upregulated under vascular stress, promotes inflammation and endothelial dysfunction.^360^ Interestingly, both the AMPK and PI3K/Akt pathways can inhibit NF-κB activation, suggesting a regulatory network that balances endothelial repair and inflammation.^361,362^ These interactions reveal the complexity of phototherapy’s impact on vascular signaling and underscore the need for targeted modulation strategies depending on the disease context.

In summary, phototherapy has significant potential in the treatment of cardiovascular diseases by targeting endothelial repair, reducing oxidative stress, and modulating inflammatory pathways. However, compared with cancer, where phototherapy aims to amplify oxidative stress to induce apoptosis, the therapeutic strategy in cardiovascular disease must balance ROS modulation to avoid further tissue injury. Therefore, antioxidant nanoparticle systems and NO-enhancing platforms take precedence in cardiovascular disease models. This distinction in ROS dynamics underscores the importance of disease-specific customization of nanoparticle design and therapeutic dosing.

### Neurodegenerative diseases

In contrast to cancer therapies that promote ROS generation and apoptosis, phototherapy in neurodegenerative diseases focuses on neuroprotection by modulating mitochondrial stability, reducing the ROS burden and preserving neuronal viability. Neurodegenerative diseases, such as Alzheimer’s disease, Parkinson’s disease and Huntington’s disease, are characterized by the progressive loss of neuronal structure and function and are often linked to protein aggregation,^363^ neuroinflammation, and neuronal apoptosis.^364^ Recent advances in phototherapy^365,366^, particularly photothermal and PBM techniques, have shown significant potential for addressing the underlying drivers of these diseases (Fig. 7 & Table 2).

#### Mechanisms affecting protein aggregation

**Fig. 8:**
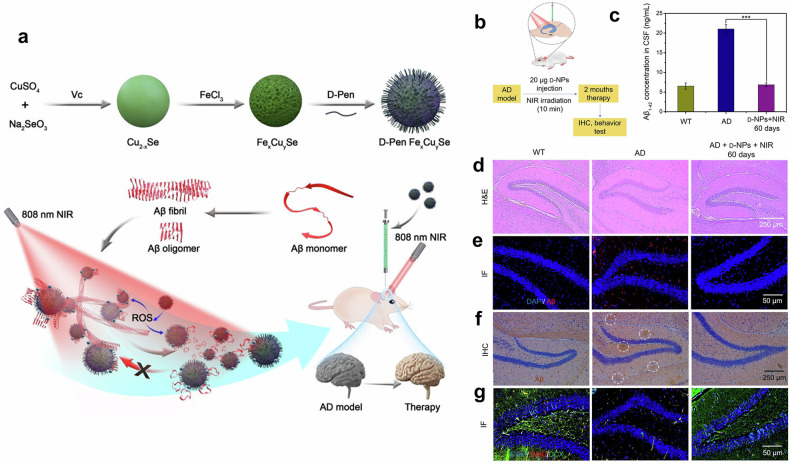
**a** Synthesis of penicillamine-functionalized FexCuySe nanoparticles and their inhibitory and disassembling effects on Aβ42 aggregation, demonstrating reduced neurotoxicity in an Alzheimer’s disease (AD) mouse model. **b** Schematic showing the administration of d-NPs in an Alzheimer’s disease (AD) mouse model. **c** Aβ42 concentration in cerebrospinal fluid following 60 days of treatment. The therapeutic effect of d-NPs combined with NIR irradiation in mitigating Aβ aggregation-induced neurotoxicity. **d** Hematoxylin and eosin (H&E) staining of the hippocampus (scale bar: 250 µm). **e** Immunostaining of Aβ42 in the hippocampus (scale bar: 50 µm). **f** Corresponding immunohistochemical images of Aβ42 in the hippocampus (scale bar: 250 µm). **g** Immunostaining for BrdU and DCX in the hippocampus (scale bar: 50 µm). Adapted with permission from^377^

One of the defining features of neurodegenerative diseases, particularly Alzheimer’s disease, is the accumulation of Aβ plaques and tau protein tangles in the brain. These aggregates disrupt neuronal communication and lead to cell death. Photothermal and PBM therapies have demonstrated promising results in targeting Aβ plaques, a hallmark of Alzheimer’s disease.^367^ The mechanisms underlying this effect involve the absorption of light by chromophores in the brain, resulting in localized heat generation or energy transfer.^368,369^ This process destabilizes the β-sheet-rich structures of amyloid plaques, promoting their disaggregation into smaller, nontoxic fragments. Furthermore, photothermal effects, such as neprilysin and insulin-degrading enzymes, which further degrade disaggregated fragments, can enhance the enzymatic activity involved in plaque clearance.^31,370^ Experimental studies have shown significant reductions in amyloid deposition in animal models following phototherapy, suggesting its potential as a noninvasive or minimally invasive treatment for Alzheimer’s disease.^371^ For example, Yan and Sasaguri *et al*. used transgenic mouse models, such as APP/PS1 mice, which are genetically engineered to develop amyloid plaques similar to those observed in Alzheimer’s disease patients.^372,373^ These mice were subjected to NIR light therapy, typically within a wavelength range of 600–900 nm, delivered via light-emitting diode (LED) arrays or laser devices. The light source is positioned transcranially, often targeting the hippocampus and cortex, regions heavily affected by amyloid deposition^374–376^ Sessions are conducted daily or on alternate days, with durations ranging from 10 to 30 min per session, depending on the study design. The energy density used is generally between 1–10 J/cm², ensuring that the light intensity is sufficient to stimulate cellular responses without causing thermal damage. Building upon these findings, recent studies have explored the integration of nanoparticles with NIR light to increase therapeutic precision and efficacy. For example, Zhang et al. developed chiral d-Fe_x_Cu_y_Se nanoparticles (d-NPs) that can interfere with Aβ42 aggregation and promote its disassembly into monomers under 808 nm NIR irradiation (Fig. 8).^377^ Compared with their l-enantiomer or Cu2-xSe analogs, these nanoparticles exhibited a stronger binding affinity for Aβ42 fibrils and generated greater levels of ROS upon light exposure. Notably, this ROS generation occurred without photothermal effects, thereby minimizing the risk of collateral tissue damage. In MN9D neuronal cells, the d-NPs significantly reduced Aβ42 adhesion to cell membranes and prevented neuron loss following short-term NIR light treatment. In vivo, d-NPs alleviated Aβ42-associated neurotoxicity and cognitive impairment in AD mouse models, suggesting promising evidence for the utility of nanoparticle-assisted phototherapy in mitigating neurodegenerative pathology. In some studies, AuNPs conjugated with Aβ-specific ligands are also injected intravenously prior to light exposure.^370,378^ These nanoparticles facilitate targeted photothermal effects, aiding in the disaggregation of amyloid plaques. Alternatively, photosensitizers such as porphyrins or chlorins are used to amplify the generation of ROS, promoting plaque clearance while minimizing damage to healthy tissues.^378,379^

**Fig. 9:**
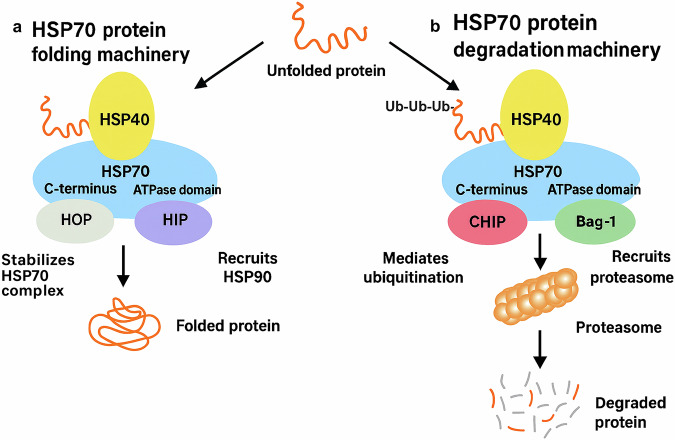
HSP70 chaperone mechanism. The fate of client proteins depends on competing interactions at the binding sites of HSP70. HOP and CHIP compete for C-terminal binding, whereas HIP and Bag-1 interact with the ATPase domain. When HIP and HOP associate with HSP70, proteins are guided toward proper folding (**a**) In contrast, CHIP and Bag-1 promote degradation through the ubiquitin‒proteasome system (**b**). HOP HSP70-HSP90 organizing protein, CHIP carboxyl terminus of HSC70-interacting protein, HIP HSP70-interacting protein, Bag-1 Bcl-2-associated athanogene-1 Adapted with permission^380^

Phototherapy modulates the expression of heat shock proteins (HSPs), which play a critical role in maintaining protein homeostasis by preventing protein misfolding and aggregation (Fig. 9).^380^ Specifically, photobiomodulation can upregulate the production of HSP70 and HSP90, which act as molecular chaperones to refold damaged proteins and facilitate the degradation of irreparably misfolded proteins via the ubiquitin‒proteasome system.^381,382^ This protective mechanism reduces the accumulation of misfolded proteins in neurodegenerative diseases, including Alzheimer’s disease and Parkinson’s disease. Additionally, the regulation of HSPs by phototherapy may also enhance cellular resilience to oxidative stress, further contributing to its neuroprotective effects.

In addition to these molecular effects, phototherapy has the potential to interact with other misfolded protein aggregates, such as tau tangles in Alzheimer’s disease or huntingtin protein aggregates in Huntington’s disease.^383,384^ Preclinical studies have explored this potential using animal models, including tauopathy mice (e.g., P301S or 3xTg-AD models) and Huntington’s disease models expressing mutant huntingtin proteins.^384–387^ For tau tangles, transcranial NIR light (600–850 nm) is delivered to the hippocampal and cortical regions of tauopathy model mice. These light exposures, typically conducted in 10–20-min sessions, 3–5 times per week, with energy densities of 5–10 J/cm², effectively reduced tau pathology. Immunohistochemical staining of phosphorylated tau and quantification of tangle density in key brain regions confirmed these effects. Behavioral tests, such as the Morris water maze and novel object recognition tests, further revealed cognitive improvements following treatment.

In Huntington’s disease models, phototherapy targets mutant huntingtin protein (mHTT) aggregates via either transcranial or systemic LED arrays.^384,388,389^ It has been shown to stimulate the heat shock response, notably by upregulating HSP70 and HSP90, which refold or degrade misfolded huntingtin proteins.^384,388,389^ These effects were validated through western blot analysis and confocal microscopy to measure aggregate reduction in the striatum, a critical brain region affected in Huntington’s disease. In vitro studies using neuronal cultures derived from these models provide further insights, showing that phototherapy enhances mitochondrial function and activates the ubiquitin–proteasome system, facilitating the clearance of tau and huntingtin aggregates. Techniques such as JC-1 (lipophilic cationic dye) staining for determining the mitochondrial membrane potential and assays for assessing proteasomal activity support these findings.

Although much of the research remains in preclinical stages, these findings suggest that phototherapy could offer a multifaceted approach to managing protein aggregation in neurodegenerative diseases.

#### Modulation of neuroinflammation

Neuroinflammation, which is primarily mediated by activated microglia, is a key contributor to the progression of various neurodegenerative disorders.^390^ Phototherapy has been shown to suppress microglial activation, thereby reducing the release of proinflammatory cytokines such as TNFα, IL-1β and IL-6.^55,391^ The light energy absorbed during phototherapy modulates intracellular signaling pathways, such as the NF-κB and MAPK pathways, which are central to inflammatory responses.^321^ By mitigating excessive microglial activity, phototherapy reduces neurotoxicity and creates a more favorable environment for neuronal survival and repair.

Astrocytes play a dual role in neuroinflammation, both exacerbating and mitigating neuronal damage, depending on their activation state.^392^ Phototherapy influences astrocytic function by promoting their transition to a neuroprotective phenotype, a phenomenon observed in both in vitro and in vivo studies.^393^ The integration of nanoparticles with phototherapy represents a promising frontier in this field, as nanoparticles can enhance precision, penetration and efficacy in targeting astrocytic pathways. While direct studies on nanoparticle-mediated phototherapy targeting astrocytes remain limited, existing research on nanoparticle-based drug delivery to astrocytes and light-based neurotherapy provides a strong rationale for their combined application. In vitro models typically use primary astrocyte cultures exposed to inflammatory stimuli, such as lipopolysaccharides (LPS), to induce a reactive proinflammatory state.^394,395^ NPs functionalized with astrocyte-targeting ligands could be introduced into these cultures to increase light absorption and enable localized activation during phototherapy. NIR light (650–850 nm) delivered at energy densities of 1–5 J/cm² has been shown to influence neuroinflammatory responses, and its interaction with nanoparticle-based systems may provide an additional level of control. Following treatment, the astrocytes are analyzed for changes in the activation state, with markers such as glial fibrillary acidic protein for astrocyte reactivity, IL-10 for anti-inflammatory signaling and brain-derived neurotrophic factor (BDNF) for neurotrophic activity measured via ELISA or Western blotting. Similarly, in vivo studies using rodent models of neuroinflammation, such as LPS-induced neuroinflammation or traumatic brain injury, could provide valuable insights into the effects of nanoparticle-mediated phototherapy.^396,397^ Systemically delivered nanoparticles can accumulate in inflamed brain regions via the EPR effect, allowing targeted phototherapy. Transcranial NIR phototherapy has been explored for its ability to modulate neuroinflammatory pathways, and when combined with nanoparticles, it could amplify therapeutic effects by facilitating controlled activation and delivery. Posttreatment analysis involves immunohistochemical staining to assess astrocytic phenotypes and evaluate reductions in proinflammatory markers (e.g., IL-1β and TNFα) and increases in anti-inflammatory and neurotrophic markers (e.g., IL-10 and BDNF).^398^

Astrocytes are also pivotal in maintaining glutamate homeostasis within the central nervous system, primarily through the uptake and release of glutamate, thereby preventing excitotoxicity, a key factor in neuronal injury during neuroinflammatory conditions.^399^ While direct studies on nanoparticle-mediated phototherapy targeting astrocytic glutamate regulation are currently limited, existing research provides a foundation for exploring this innovative approach. For example, selenium nanorods have been shown to induce calcium signaling in cortical astrocytes, suggesting potential pathways for modulating astrocytic activity.^400–402^ The incorporation of nanoparticles into phototherapy could enhance their targeting ability and efficacy in modulating astrocytic responses. This combined approach may promote the transition of astrocytes to a neuroprotective phenotype, encouraging anti-inflammatory cytokine secretion, neurotrophic factor production and effective glutamate regulation. Such strategies hold promise for addressing neuroinflammatory conditions and supporting neuronal recovery through precise modulation of astrocytic pathways.^403,404^ Future research could focus on developing nanoparticles functionalized with ligands specific to astrocytic glutamate transporters, such as GLT-1 and GLAST. These nanoparticles could be designed to enhance light absorption and enable localized activation during phototherapy. The subsequent application of NIR light could facilitate deeper penetration and targeted activation, potentially overcoming the limitations of traditional phototherapy. By advancing these strategies, we can better understand and harness the role of astrocytes in CNS health and disease, paving the way for novel therapeutic interventions in neuroinflammatory and neurodegenerative disorders.

In neurodegenerative disorders, multiple signaling pathways, such as the Nrf2, PI3K/Akt and AMPK pathways, affect neuronal survival but diverge in how they regulate redox balance and autophagy.^405,406^ For example, Nrf2 activation leads to antioxidant enzyme expression, whereas PI3K/Akt promotes neuronal survival via Bcl-2 family proteins.^407,408^ On the other hand, AMPK activation enhances autophagy, which can either protect against or exacerbate degeneration depending on the disease stage. The subtle yet critical differences in the roles of these pathways highlight the complexity of designing phototherapy regimens that avoid tipping the balance toward neurotoxicity.

#### Prevention of neuronal apoptosis

Neuronal apoptosis, largely driven by mitochondrial dysfunction, is a key contributor to neurodegenerative conditions such as Alzheimer’s, Parkinson’s and Huntington’s disease.^409^ This process is exacerbated by oxidative stress, energy deficits and the activation of apoptotic pathways, ultimately leading to neuronal death. PBM has demonstrated neuroprotective potential by stabilizing mitochondrial function, improving ATP production, reducing oxidative stress and maintaining the mitochondrial membrane potential.^410^ These effects are primarily mediated through the activation of cytochrome c oxidase, a key enzyme in the mitochondrial respiratory chain that absorbs specific wavelengths of light, thereby improving cellular energy metabolism and preventing the release of proapoptotic factors such as cytochrome c.^411^

An emerging avenue for enhancing the precision and efficacy of phototherapy involves nanoparticle-mediated light delivery.^401^ Functionalized nanoparticles, such as AuNPs and upconversion nanoparticles, have been explored for their ability to amplify photothermal and PBM effects at the mitochondrial level.^412,413^ These nanoparticles act as light transducers, improving energy absorption and enabling deeper tissue penetration, thereby overcoming some of the limitations of conventional PBM. In preclinical models, nanoparticles designed to localize to neurons have enhanced mitochondrial stabilization following NIR light exposure, reducing apoptotic signaling and preserving neuronal viability.^414^

In addition to mitochondrial stabilization, nanoparticle-mediated phototherapy has the potential to modulate neurotrophic signaling, a crucial factor in neuronal survival and synaptic plasticity. Studies have shown that PBM upregulates BDNF and nerve growth factor (NGF), both of which promote neuronal growth and survival by activating the receptors TrkB and TrkA.^415,416^ The incorporation of nanoparticles into this framework could allow for increased targeted activation of neurotrophic pathways, potentially leading to increased sustained neuroprotection in neurodegenerative models.

Experimental studies have also shown that phototherapy can prevent apoptosis by reducing the accumulation of toxic protein aggregates, such as Aβ and alpha-synuclein, which are known to trigger apoptotic pathways.^417^ Furthermore, the anti-inflammatory effects of phototherapy indirectly contribute to preventing apoptosis by reducing the levels of proapoptotic signals associated with chronic inflammation.

Despite the therapeutic potential of nanoparticle-assisted phototherapy for treating neurodegenerative diseases, the unique vulnerability of brain tissue has raised serious concerns. Even minimal off-target accumulation can induce neurotoxicity, trigger neuroinflammation, or impair synaptic function, potentially leading to irreversible outcomes.^418^ To mitigate these risks, several strategies are being explored. These include the development of ultrasmall, biodegradable nanoparticles that can be efficiently cleared from the central nervous system;^419^ the use of brain-targeting ligands (e.g., Tf, RVG peptides) to enhance BBB selectivity;^420^ and the engineering of stimuli-responsive nanocarriers that are activated only under tightly controlled light or enzymatic conditions.^421^ Furthermore, real-time imaging and theranostic platforms can help monitor biodistribution and reduce unintentional exposure.^422^ These approaches are critical for ensuring both efficacy and safety in the clinical translation of brain-targeted phototherapy systems.

While phototherapy in cancer emphasizes apoptosis through ROS overload, in neurodegenerative conditions, the therapeutic window for ROS is narrow because of the heightened sensitivity of neuronal tissues. Here, the strategy shifts toward fine-tuning oxidative stress, promoting autophagy and protecting mitochondrial integrity. NPs for neurological applications are therefore often optimized for controlled ROS modulation, BBB penetration and minimal off-target effects. This illustrates a critical divergence in therapeutic design and signaling emphasis between oncology and neurology.

### Metabolic disorders and autoimmune diseases

While phototherapy in oncology relies on triggering immunogenic cell death to activate antitumor immunity, its application in autoimmune conditions involves suppressing proinflammatory cytokines and modulating immune cell polarization to restore immune balance. Phototherapy has emerged as a promising noninvasive intervention for the management of metabolic disorders, such as type 2 diabetes, obesity and nonalcoholic fatty liver disease, as well as autoimmune diseases, including RA, multiple sclerosis and systemic lupus erythematosus (SLE).^423–428^ By targeting critical cellular processes, including oxidative stress, inflammation, energy metabolism and immune regulation, phototherapy offers a multifaceted approach to alleviating the symptoms and progression of these complex conditions. Phototherapy offers a novel therapeutic approach with wide-ranging implications by modulating signaling pathways, regulating the immune system and enhancing systemic metabolic processes (Fig. 7 & Table 2).

#### Interactions with insulin signaling pathways

One of the primary mechanisms by which phototherapy impacts metabolic disorders, such as type 2 diabetes, is by enhancing insulin sensitivity.^425,429^ Insulin resistance is a hallmark of type 2 diabetes; therefore, glucose uptake is impaired by insulin-responsive tissues, such as skeletal muscle and adipose tissue.^430^ Phototherapy stimulates the translocation of glucose transporter type 4 (GLUT4) to the cell membrane, a critical step in glucose uptake in insulin-responsive tissues such as skeletal muscle and adipose tissue.

The molecular mechanisms underlying this effect involve the activation of key metabolic pathways, such as the adenosine monophosphate-activated protein kinase (AMPK) and PI3K-Akt signaling pathways.^431^ AMPK, often referred to as a cellular energy sensor, is activated in response to changes in energy balance and plays a pivotal role in glucose and lipid metabolism. Phototherapy-induced activation of AMPK facilitates the translocation of GLUT4, thereby promoting glucose uptake and utilization in skeletal muscle and adipose tissues.^432^ Similarly, activation of the PI3K-Akt signaling cascade enhances insulin receptor sensitivity, further improving glucose homeostasis.^431^ Together, these pathways contribute to a reduction in blood glucose levels, mitigating hyperglycemia and its associated complications.

Phototherapy also exerts antioxidative and anti-inflammatory effects, which are crucial in mitigating diabetic complications. By decreasing the production of ROS and enhancing the activity of antioxidant enzymes such as SOD and catalase, phototherapy reduces oxidative stress in diabetic tissues.^433^ Additionally, it modulates the levels of proinflammatory cytokines, such as TNFα and IL-6, which are elevated in insulin-resistant states.^434^ This dual action helps to preserve cellular integrity, improve insulin signaling and protect against the progression of diabetes-related tissue damage.

#### Modulation of autoimmune pathways

Autoimmune diseases, including RA and SLE, are characterized by dysregulated immune responses that lead to chronic inflammation and tissue damage. Phototherapy has demonstrated significant potential in modulating autoimmune pathways, making it a promising therapeutic approach for these conditions. One of the key mechanisms of phototherapy in autoimmune diseases is its ability to influence T-cell differentiation.^2^ Autoimmune pathology is driven primarily by proinflammatory T-helper 1 (Th1) and T-helper 17 (Th17) cells, which produce cytokines such as interferon-gamma (IFN-γ), TNFα and IL-17. These cytokines exacerbate inflammation and tissue damage. Phototherapy selectively suppresses the activity of Th1 and Th17 cells while promoting the differentiation of T-helper 2 (Th2) cells, which secrete anti-inflammatory cytokines such as IL-4 and IL-10.^435,436^ This shift in T-cell balance reduces the levels of cytokines such as IFN-γ, TNF-α and IL-17, which are implicated in autoimmune inflammation, thereby alleviating disease symptoms and progression.^435,437,438^ Animal models such as collagen-induced arthritis for RA and MRL/lpr mice for SLE are used to evaluate the therapeutic effects of phototherapy.^114,293,439^ In these studies, functionalized nanoparticles were employed to enhance the absorption and targeting of NIR light. These nanoparticles are conjugated with ligands or antibodies to ensure selective accumulation in inflamed tissues, such as affected joints in RA or lupus-involved organs such as the kidneys. NIR light (650–850 nm) is delivered transdermally via a laser, with energy densities ranging from 5 to 10 J/cm², for 10–20 min per session, and it is applied 3–5 times weekly. Following treatment, cytokine profiling via ELISA and flow cytometry revealed reduced levels of IFN-γ, TNF-α and IL-17 alongside increased levels of IL-4 and IL-10. Immunohistochemical analysis of affected tissues demonstrated a reduction in inflammatory infiltrates, whereas behavioral assessments, such as arthritis scores and proteinuria measurements, confirmed a significant improvement in disease control.

Another critical aspect of phototherapy’s immunomodulatory effects is its impact on Tregs. Tregs play a pivotal role in maintaining immune tolerance and preventing autoimmunity.^440^ Phototherapy has been shown to promote the expansion and functional activity of Tregs by upregulating key transcription factors such as FoxP3.^441–443^ This increase in Treg activity suppresses autoimmune responses and improves immune homeostasis.^444^

In addition to T-cell modulation, phototherapy also reduces the activation and proliferation of autoreactive B cells, which contributes to autoantibody production in diseases such as SLE.^445–447^ By suppressing these pathogenic immune responses, phototherapy could provide long-term immunomodulation, reduce disease flares and improve overall treatment outcomes.

#### Systemic metabolic pathways

Phototherapy exerts systemic effects that extend beyond localized treatment, influencing overall energy metabolism and metabolic health. A central mechanism through which phototherapy improves systemic metabolic pathways is its ability to enhance mitochondrial function.^425^ Mitochondria, the powerhouses of the cell, play a critical role in energy production and are often impaired in metabolic syndromes such as obesity and type 2 diabetes.^448^ Phototherapy activates cytochrome c oxidase, which, in turn, increases ATP production, restoring the cellular energy balance and improving metabolic efficiency.^449,450^ Preclinical studies using high-fat diet-induced obese mice and Zucker diabetic rats have shown that transdermal phototherapy applied to metabolically active tissues, such as skeletal muscle and adipose tissue, significantly improves metabolic parameters.^451–453^ Conventional phototherapy, typically delivered at energy densities of 3–10 J/cm² for 10–20 min per session over 2–4 weeks, has been shown to improve glucose tolerance, insulin sensitivity and systemic inflammatory markers in animal models. Despite these promising results, the efficiency of phototherapy is often limited by tissue penetration depth and nonspecific energy dispersion, particularly in metabolically dense tissues such as visceral fat. A compelling avenue for enhancing the precision and efficacy of phototherapy is the integration of nanoparticle-based light delivery systems. While direct studies in metabolic disorders remain scarce, nanoparticle-mediated approaches have been widely explored in cancer therapy and neuroscience, demonstrating their potential to increase phototherapy specificity and intracellular effects. NPs can be functionalized to target mitochondria, the central hub of metabolic regulation, thereby amplifying the effects of phototherapy at the cellular level. Furthermore, nanoparticles engineered for selective uptake by skeletal muscle or adipose tissue could provide targeted modulation of metabolic pathways, enhancing glucose uptake, fatty acid oxidation and even the browning of white adipose tissue, an essential process for increasing thermogenic activity and energy expenditure. An additional advantage of nanoparticle-assisted phototherapy is its potential to modulate inflammatory signaling, a key contributor to metabolic disease progression. Phototherapy has already been shown to reduce the levels of proinflammatory cytokines, such as TNFα and IL-6, which impair insulin signaling and promote metabolic dysfunction. By integrating nanoparticles, phototherapy could be optimized to target inflammatory microenvironments, further enhancing its therapeutic impact.

Adipokines, such as adiponectin and leptin, play a central role in regulating systemic metabolic homeostasis.^454^ Adiponectin enhances insulin sensitivity, reduces inflammation and improves lipid metabolism, whereas leptin regulates appetite and energy expenditure. Dysregulation of these adipokines is a common feature of metabolic disorders. Phototherapy influences the secretion of these adipokines by modulating the function of adipocytes and other metabolic tissues.^455^ Increased adiponectin levels enhance insulin sensitivity and anti-inflammatory responses, whereas normalization of leptin levels regulates appetite and energy expenditure. These effects contribute to improved metabolic health and reduced risks associated with obesity and metabolic disorders.

In addition to its effects on energy metabolism and adipokines, phototherapy also improves vascular function, which is often compromised in metabolic disorders. By enhancing endothelial function and reducing vascular inflammation, phototherapy improves blood flow and oxygen delivery to tissues, supporting metabolic health and preventing complications such as cardiovascular disease.^456^

The ability of phototherapy to modulate critical pathways involved in metabolic disorders and autoimmune diseases underscores its potential as a versatile and noninvasive therapeutic approach. By addressing the root causes of these conditions, including oxidative stress, inflammation and immune dysregulation, phototherapy offers a comprehensive strategy for improving patient outcomes. As research continues to uncover the molecular mechanisms and clinical applications of phototherapy, its role in the management of chronic diseases is poised to expand, offering new hope to patients worldwide.

Key signaling pathways modulated in inflammatory diseases, such as NF-κB, STAT3 and TGF-β, all influence immune cell behavior but exhibit unique patterns of regulation upon phototherapy.^457^ NF-κB suppression generally reduces the production of proinflammatory cytokines (e.g., IL-1β and TNF-α), whereas STAT3 inhibition alters T-cell differentiation, particularly by reducing Th17 responses.^458^ In contrast, TGF-β modulation can have dual effects: dampening inflammation but also contributing to tissue fibrosis.^459^ These observations underscore the need for pathway-specific tuning when phototherapy is applied in immune-mediated disorders, as broad-spectrum modulation may inadvertently promote immunopathology.

In autoimmune diseases, phototherapy often targets immune reprogramming, favoring anti-inflammatory signaling over proinflammatory signaling. This differs markedly from cancer, where immune activation is desirable. For example, the modulation of macrophage polarization (M1/M2) in both disease types serves different goals: tumor suppression in cancer versus inflammation resolution in autoimmunity. This functional inversion highlights how identical molecular targets can play opposing roles, emphasizing the need for disease-specific signal mapping when designing nanoparticle-based phototherapies.

## Clinical applications

**Fig. 10:**
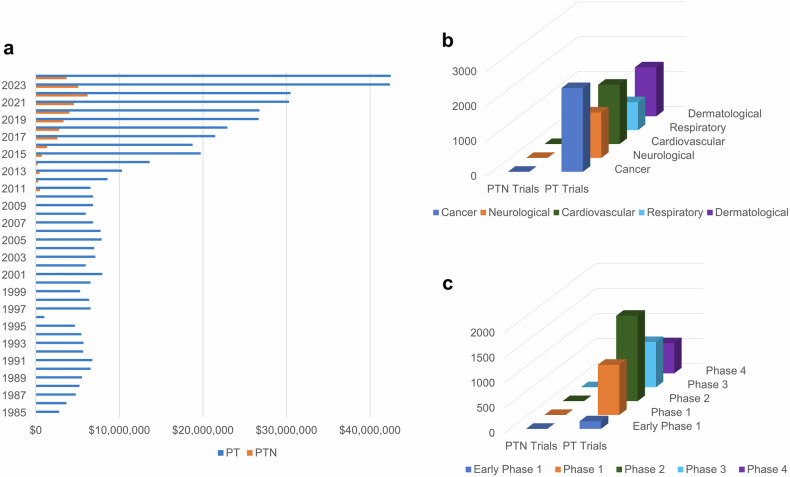
Funding and distribution of phototherapies (PTs) and nanoparticle-based phototherapies (PTNs) across time, medical conditions and clinical trial phases. **a** The chart illustrates the funding trends for PT and PTN over time, showing a significant increase in recent years, with 2023 having the highest funding. PT trials dominate funding allocations, whereas PTN studies have a smaller but growing interest. **b** The distribution of PT and PTN trials across various medical conditions, with cancer having the highest number of PT trials, followed by cardiovascular and dermatological conditions. **c** The breakdown of trials by clinical phase indicates that Phase 2 has the highest number of PT trials, followed by Phase 3 and Phase 4, whereas there are fewer early Phase 1 and Phase 1 trials. Source: Data generated via information from clinicaltrials.gov and the NIH Reporter

The clinical translation of phototherapy represents a significant leap forward in the treatment of various diseases, leveraging advances in nanotechnology and optical systems. From FDA-approved drugs to promising findings from ongoing clinical trials, the use of phototherapy continues to expand in clinical settings (Fig. 10).

### FDA-approved drugs and their mechanisms

Phototherapy drugs approved by the FDA underscore the transformative potential of this technology, particularly in diseases such as cancer. Despite the vast promise demonstrated by preclinical and clinical studies, only a few drugs, such as Photofrin^®^ and Visudyne^®^, have gained FDA approval. This limited success reflects the complex regulatory and developmental hurdles associated with phototherapy. The intricate interplay of light activation, photosensitizer pharmacokinetics and treatment precision introduces layers of complexity in standardizing clinical protocols. Regulatory agencies such as the FDA demand rigorous evidence of safety, efficacy and reproducibility, criteria that can be difficult to meet given the variability in light delivery systems and the dependence on patient-specific factors such as tissue oxygenation and light penetration.

#### Photofrin^®^ (Porfimer sodium)

Photofrin^®^ is a hematoporphyrin derivative and one of the first-generation photosensitizers used in PDT.^460,461^ It consists of a complex mixture of oligomeric porphyrins obtained through acidification and purification of hematoporphyrin. This structural complexity enhances its light absorption efficiency and singlet oxygen production, making it effective in PDT applications. The commercial formulation is provided as a sterile lyophilized powder reconstituted in 5% dextrose or saline for intravenous administration.^462^ The standard clinical dose is 1–2 mg/kg, followed by light activation at 630 nm after a 24–48 h drug-to-light interval to allow selective accumulation in tumor tissues.

Photofrin has received FDA approval for multiple indications.^460^ It is widely used as a palliative treatment for esophageal cancer, particularly for obstructive tumors where conventional therapies are not viable.^463^ In early-stage non-small cell lung cancer and bronchial dysplasia, PDT with Photofrin has been shown to be effective in eradicating premalignant and malignant lesions.^464,465^ Additionally, it is being investigated in clinical trials for nonmuscle invasive bladder cancer and cervical cancers associated with HPV infections.^466,467^ Despite these applications, its clinical use remains limited by certain drawbacks, including prolonged skin photosensitivity, which can persist for 4–6 weeks post-treatment, restricting patient mobility.^47^ Moreover, the 630 nm activation wavelength provides limited tissue penetration (approximately 4–5 mm), making it less effective for deeply seated tumors.^468^ Off-target accumulation in some normal tissues can also result in unintended side effects.^469^ To address these challenges, emerging formulations and advanced PDT strategies are being explored. Nanoparticle-encapsulated Photofrin has shown promise in enhancing tumor retention, reducing systemic toxicity and improving treatment precision.^470^ Additionally, combination therapies that integrate PDT with immunotherapy or chemotherapy are being investigated to increase the overall therapeutic efficacy.^471^ Another promising approach is the application of two-photon excitation PDT, which employs NIR light to achieve deeper tissue penetration while minimizing phototoxic side effects.^472^ The role of nanoparticles in PDT continues to expand, with the potential for actively targeted nanocarriers that improve tumor selectivity while modulating the tumor microenvironment to enhance ROS-mediated cytotoxicity.

#### Visudyne^®^ (Verteporfin)

Verteporfin, marketed as Visudyne^®^, is a benzoporphyrin derivative used as a photosensitizer in PDT, particularly for ocular conditions.^473,474^ It is a 1:1 mixture of two regioisomers, benzoporphyrin derivative monoacid A and benzoporphyrin derivative monoacid B, synthesized through the Diels–Alder reaction between protoporphyrin IX dimethyl ester and acetylene dicarboxylic acid dimethyl ester, followed by hydrolysis to yield the active compound. The formulation is provided as a sterile, lyophilized powder, which is reconstituted with sterile water for injection to a concentration of 2 mg/mL. The standard dose is 6 mg/m² body surface area and is administered via intravenous infusion over 10 min.^475,476^ Following administration, light at 689 nm is applied to the target area, typically 15 min after the start of the infusion.

Upon intravenous administration, verteporfin binds primarily to low-density lipoproteins (LDLs) in the plasma, facilitating its transport to target tissues. In the context of age-related macular degeneration, verteporfin accumulates preferentially in neovascular endothelial cells because of its elevated LDL receptor expression.^477^ Activation with 689 nm light in the presence of oxygen leads to the generation of singlet oxygen and other ROS, causing localized damage to the neovascular endothelium and subsequent vessel occlusion. This process effectively reduces choroidal neovascularization, thereby stabilizing or improving vision in affected patients.

Clinically, verteporfin is approved for the treatment of predominantly classic subfoveal choroidal neovascularization due to age-related macular degeneration, pathological myopia, or presumed ocular histoplasmosis.^478^ Its use has significantly helped in the management of these conditions by offering a targeted, minimally invasive treatment option.

Despite its efficacy, verteporfin-driven PDT has limitations, including the need for precise light delivery and the potential for photosensitivity reactions, necessitating patient avoidance of direct sunlight and bright indoor lighting for 48 h post-treatment.^479^ To address these challenges, research into nanoparticle-based delivery systems for verteporfin is ongoing.^480^ The encapsulation of verteporfin in liposomes, polymeric nanoparticles, or other nanocarriers aims to increase its solubility, improve its selective accumulation in target tissues and reduce off-target effects.^481^ These advancements hold promise for increasing the therapeutic index of verteporfin and expanding its applications beyond ophthalmology to include oncology and other fields.

The approval of only two phototherapy drugs, despite decades of research, highlights the critical need for advancements in nanoparticle-based phototherapy. Second-generation PSs, often incorporating nanotechnology, offer superior tumor selectivity, reduced side effects and enhanced light absorption properties. However, their journey from the laboratory to the clinic is often stalled by the high costs of development, stringent regulatory requirements and the challenges of demonstrating superiority over existing treatments in large-scale trials. The evolving field of nanoparticle-based phototherapy holds immense promise, not only in cancer but also in other diseases.

### Insights from ongoing clinical trials

**Fig. 11:**
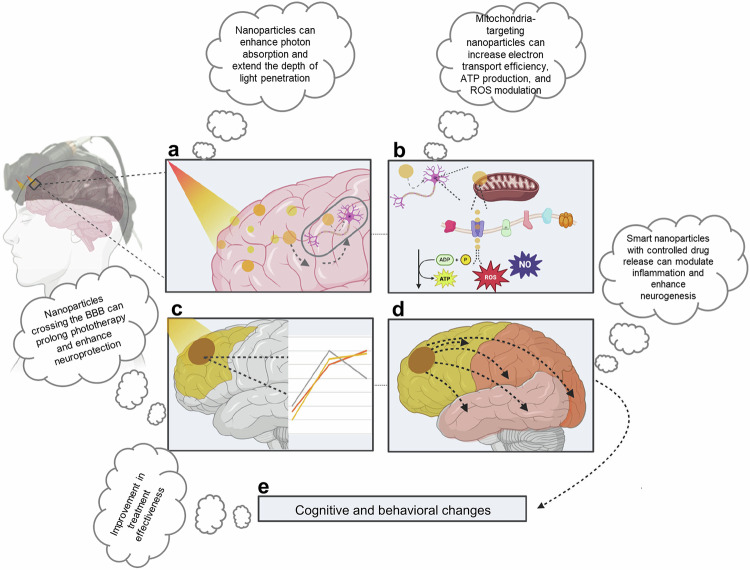
Mechanism of t-PBM and perspective (in bubbles) of nanomedicines in phototherapy-driven brain energy metabolism. **a** Photons penetrate the skull, with an 808 nm wavelength reaching 40–50 mm into the cortex. Up to 5% of photons may be refracted, while the rest may enter the cerebral cortex. **b** Neurons absorb these photons, primarily through cytochrome c oxidase in the mitochondrial membrane, enhancing the electron transport chain, increasing ATP production, modulating reactive oxygen species and releasing nitric oxide. **c** These changes occur in t-PBM-irradiated cerebral regions and influence intrinsic brain activity before, during and after irradiation. **d** Downstream effects include transcription factor activation and responses beyond the irradiation site. **e** These mechanisms contribute to behavioral and clinical outcomes, such as mood and cognitive improvements. Adapted and modified with permission from^544^

Phototherapy-based clinical trials have demonstrated significant potential in addressing unmet medical needs, particularly in areas such as oncology, dermatology, ophthalmology and even autoimmune disorders. By leveraging advancements in light delivery systems and integrating nanotechnology, these therapies enable unparalleled precision and selectivity. For example, PDT combined with nanoparticles has been shown to enhance light absorption and extend drug retention in tumor tissues, overcoming the limitations of first-generation photosensitizers.^37^ Similarly, nanoparticle-driven PTT offers precise thermal ablation of cancer cells with minimal damage to surrounding healthy tissues. While the field of nanoparticle-based phototherapy has been widely explored in cancer treatment, emerging perspectives suggest that nanoparticles could significantly enhance the effects of phototherapies in different disorders as well as transcranial PBM (t-PBM) by improving light delivery, cellular uptake and the mitochondrial response (Table 3). Figure 11 outlines the molecular mechanisms of t-PBM and highlights areas where nanoparticles could further optimize its therapeutic effects.

**Table 3:** Summary of clinical trials, listing nanoparticle types, targeted pathways and disease applications

Clinical trial numbers	Nanoparticle type	Mechanisms of action	Disease applications	Type of light	Key findings/insights
NCT05736224	Bioadhesive nanoparticles	UV radiation protection	Skin cancer	UV light	Protection against UVR-induced cellular damage
NCT05666011	Silver nanoparticles	Light activation	Keratosis pilaris	Intense pulsed light	Enhanced efficacy for skin condition treatment
NCT01270139	Silica-AuNPs	Photothermal therapy	Atherosclerosis	NIR laser	Feasibility of reducing cardiovascular plaque
NCT06761248	Starch nanoparticles	Diagnostic fluorescence	Dental caries	Fluorescence	Effective diagnostic tool for early dental caries
NCT02353611	Titanium oxide nanoparticles	Oxidative whitening mechanism	Tooth bleaching	Blue hybrid light and infrared laser	Improved tooth whitening with reduced side effects
NCT06533150	PLGA nanoparticles	Tissue regeneration	Craniofacial bone defects	LED	Biocomposite for bone regeneration
NCT00389714	Antibacterial nanoparticles	Antimicrobial restorative material	Dental caries	LED	Long-term durability of nanocomposite restorative materials
NCT06601972	Nanohybrid resin composite	Multi-Ion release	Dental caries	LED	Prevents demineralization and enhances enamel resistance
NCT06399328	Silver nanoparticles	Diagnostic biomarkers	Coronary artery disease	NIR laser	Novel diagnostic insights for cardiovascular risk assessment
NCT04738604	Nano ceramic resin	Restorative material properties	Class I dental caries	LED	Improved wear resistance of nanocomposite restorations

Although current t-PBM studies focus primarily on direct photonic stimulation, incorporating nanoparticle-mediated approaches could revolutionize the field. Nanoformulations designed to increase light absorption, facilitate targeted delivery, or coadminister neuroprotective agents could overcome existing limitations, such as limited penetration depth, rapid photon dissipation and variability in patient response.

Several ongoing clinical trials continue to explore the role of nanoparticle-based phototherapy across various medical fields. In oncology, MRI/US fusion imaging trials (**NCT04240639, NCT02680535**) have assessed the use of gold-based AuroShell nanoparticles for PTT in prostate cancer, whereas a plasmonic nanophotothermal therapy trial for atherosclerosis (**NCT01270139**) has investigated the use of laser-activated silica-AuNPs to break down arterial plaques. In dermatology, the keratosis pilaris treatment trial (**NCT05666011**) examines M22 intense pulsed light therapy, whereas the minoxidil delivery trial for alopecia areata (**NCT05587257**) explores fractional CO_2_ laser-assisted nanoparticle-enhanced drug delivery.

In addition to cancer and dermatology, nanoparticle-enhanced phototherapy has also been explored in the context of infectious disease management. The photodisinfection with chitosan nanoparticles trial (**NCT06523244**) investigated nanoparticle-enhanced ICG photodegradation, which disrupts bacterial biofilms and enhances immune responses in periodontal disease. Moreover, in dentistry, the hydrogen peroxide tooth bleaching trial (**NCT02353611**) examines how laser-activated nanoparticles can enhance stain breakdown while preserving enamel integrity.

These studies underscore the growing role of nanoparticle-based phototherapy in modern medicine. While t-PBM has demonstrated promising outcomes in neurological conditions, the integration of nanoparticle-based strategies could further enhance light-based brain stimulation. Whether through the use of BBB-penetrating nanoparticles, photoresponsive nanomaterials, or neuroprotective nanoformulations, the convergence of nanotechnology and phototherapy has the potential to expand therapeutic applications, improve precision and optimize patient outcomes in neurology and beyond.^482–484^ One key observation emerging from clinical trials is the dual role of phototherapy in not only destroying diseased cells but also modulating the microenvironment.^268^ This includes promoting the modulation of immune responses, which can enhance the overall efficacy of treatments. In dermatology, phototherapy has been shown to be highly effective in conditions such as psoriasis and vitiligo by targeting overactive immune pathways without systemic immunosuppression.^485,486^

### Current challenges and strategies for overcoming barriers

Despite its success, phototherapy faces many challenges that hinder its widespread adoption. Addressing these barriers requires a multidisciplinary approach that integrates nanotechnology, pharmacology and clinical sciences.

#### Safety and biocompatibility

The long-term safety of nanoparticles remains a concern, particularly regarding their retention in tissues and potential toxicity.^487^ To address this, researchers are developing biodegradable nanoparticles, such as polymeric micelles, nanoparticles and liposomes, that degrade into nontoxic metabolites while maintaining therapeutic efficacy.^39,488,489^ Furthermore, the immunogenicity of nanoparticle components, possible off-target effects and cumulative toxicity from repeated administrations are being actively studied in preclinical models.^490,491^ Comprehensive safety profiling, including long-term biodistribution, immunotoxicity and genotoxicity studies, is essential before clinical translation. Stimuli-responsive designs also enable controlled disassembly under specific intracellular conditions, such as acidic pH or redox gradients, thereby improving therapeutic precision and minimizing unintended effects.^492^

Additionally, the stability of nanoparticles is essential for preserving their functional and therapeutic efficacy.^493^ Factors such as size and surface energy affect their aggregation tendencies, which in turn influences circulation time and bioavailability.^494^ Stabilizers such as PEG, surfactants, or dendrimers improve colloidal stability, minimize RES clearance and prolong the systemic half-life.^495,496^ However, anti-PEG antibodies (APAs) can accelerate the clearance of PEGylated drugs at high titers, necessitating the consideration of APA levels during therapy design.^497^

#### Targeting and precision

The ability to achieve precise delivery of phototherapy agents in diseased tissues without affecting healthy cells is a significant hurdle.^498^ Recent advances include the use of surface-functionalized nanoparticles with tumor-specific ligands, such as folic acid, TCRs, or antibodies, ensuring targeted accumulation.^499,500^ Light delivery systems, such as fiber-optic catheters, are also being developed to increase spatial precision in deep-seated tissues.^501^

#### Scalability, cost effectiveness and quality control

The complexity of synthesizing nanoparticles and developing light-based systems makes phototherapy expensive. Researchers are exploring cost-effective methods, such as the green synthesis of nanoparticles using plant extracts and the integration of phototherapy with existing clinical tools, to lower costs.^488^ However, translating laboratory-scale synthesis protocols to industrial-scale production remains a significant challenge.^502^ Batch-to-batch variability, the need for good manufacturing practice (GMP) compliance and stability during storage and transport add further complexity.^503^ Establishing standardized manufacturing workflows and scalable purification methods will be critical for widespread clinical implementation.

In addition, quality control protocols must be developed to monitor parameters such as the particle size distribution, surface chemistry, drug loading efficiency and photoresponsiveness.^504^ The integration of phototherapy components (e.g., light delivery systems) into standardized clinical kits requires streamlined assembly, validation and regulatory approval under stringent GMP conditions. Addressing these manufacturing bottlenecks is crucial for reliable and reproducible production, which will ultimately support broader clinical translation and commercialization of nanoparticle-based phototherapies.

#### Penetration depth of light

The limited penetration of light, especially in PDT and PTT, limits their use, primarily to superficial or easily accessible tissues.^505,506^ Recent advances in the development of photothermal and photodynamic agents responsive to NIR-II have shown substantial promise for overcoming this limitation.^507–509^ The NIR-II range offers reduced light scattering, lower tissue absorption and minimal autofluorescence, enabling deeper tissue penetration and improved spatial resolution for imaging and therapy.^510^ Nanoparticles engineered to absorb and operate efficiently within this window, such as rare-earth doped nanoparticles^511^, single-walled carbon nanotubes^512^ and semiconducting polymer nanostructures^513^, are emerging as next-generation platforms for deep-tissue phototherapy.

Advanced phototherapeutic systems increasingly integrate external stimuli, such as magnetic fields and ultrasound, to enhance tumor targeting and improve therapeutic precision.^514^ Magnetic stimuli-responsive nanoparticles, such as iron oxide/gold-loaded porous silicon hybrids, enable magnetically guided delivery, photothermal heating and image-guided therapy.^515^ Ultrasound facilitates nanoparticle extravasation through acoustic cavitation and enhances local drug release.^516^ Despite deeper penetration offered by magnetic or ultrasound methods, phototherapy remains clinically dominant due to its controllability, spatial precision and regulatory familiarity (Fig. 3).^517^

#### Tumor microenvironmental barriers

Solid tumors present unique biological barriers that hinder the efficacy of nanoparticle-based phototherapy. One major challenge is hypoxia in the tumor microenvironment, which severely limits the effectiveness of PDT by reducing oxygen availability for ROS generation.^518^ To overcome this, oxygen-releasing nanoparticles and oxygen-carrying agents such as perfluorocarbon emulsions have been investigated for their ability to increase intratumoral oxygenation.^519^ In addition, the dense and fibrotic extracellular matrix (ECM) acts as a physical barrier that restricts nanoparticle diffusion and impairs light penetration.^498^ NPs engineered with ECM-degrading enzymes, such as collagenase or hyaluronidase, have shown promise in improving tissue penetration.^520^ Other complementary strategies include designing size-shrinkable nanoparticles that respond to acidic pH or enzyme activity in tumors to enhance penetration. For example, liposomes in the 70–200 nm range exhibit optimal uptake, whereas RES-modulating agents such as GM1 can extend the circulation time.^521^ Furthermore, pH-responsive nanocarriers allow selective payload release under acidic conditions.^522^ Active targeting via ligands, such as antibodies, peptides, or aptamers, facilitates selective binding to overexpressed tumor receptors. Finally, redox-responsive designs and multistage release systems, such as irinotecan-quinine nanodrugs, show promise in overcoming multidrug resistance by leveraging tumor-specific glutathione levels for targeted intracellular drug activation.^523^ Stromal cells, including cancer-associated fibroblasts, can also contribute to therapy resistance by remodeling the ECM and promoting immune suppression. Phototherapy strategies that cotarget stromal components or modulate stromal–immune interactions are emerging as effective approaches to overcome these barriers.^441^ Moreover, understanding the dynamic crosstalk between tumor cells and stromal elements can guide the rational design of combination therapies to improve phototherapy outcomes.^524^

#### Regulatory and clinical adoption barriers

Compared with conventional drugs, phototherapy systems incorporating nanoparticles face additional regulatory scrutiny because of their hybrid nature and novel mechanisms of action.^525^ Regulatory approval requires extensive characterization of physicochemical properties, reproducibility and long-term safety data. Moreover, the lack of unified guidelines for phototherapeutic nanomedicines across global regulatory bodies (e.g., the US Food and Drug Administration and the European Medicines Agency) complicates the approval process. Clinically, limited awareness among healthcare providers, a lack of standardized treatment protocols and insufficient long-term outcome data impede adoption. Early-phase clinical trials remain sparse, and more robust multicenter studies are needed to establish efficacy, safety and optimal dosing regimens.^526^ Bridging the translational gap will also require the integration of phototherapy systems into existing clinical workflows, supported by evidence-based guidelines and real-world data.

## Conclusion and future prospects

The integration of multifunctional nanoparticles has significantly increased the precision and therapeutic scope of phototherapy. These nanomaterials enable tunable light absorption, improved tissue penetration and codelivery of multiple therapeutic agents, broadening the clinical applications of nanoparticle-based phototherapy beyond oncology to include cardiovascular diseases, inflammatory disorders and antimicrobial therapies. However, despite significant advancements, there remains a vast potential for further optimization.

Nanoparticle-based phototherapy has emerged as a transformative modality for the treatment of cancer and other chronic diseases, leveraging the precise interaction between light and engineered nanomaterials to achieve highly targeted, minimally invasive therapeutic effects. By modulating key molecular mechanisms, such as ROS generation and scavenging, apoptotic pathway regulation and immune system activation, this approach has demonstrated remarkable efficacy in both preclinical and clinical settings. The ability of phototherapy to selectively target diseased tissues while minimizing damage to healthy cells remains one of its most compelling advantages. In particular, PDT-induced ROS generation disrupts cellular homeostasis, leading to apoptosis, whereas PTT-induced hyperthermia irreversibly damages cellular structures and activates stress‒response pathways that amplify immune responses. Additionally, the capacity to trigger ICD provides dual benefits, eliminating primary tumors while also eliciting systemic antitumor immunity.

Future research should focus on identifying novel molecular targets and pathways to improve therapeutic outcomes. For example, targeting hypoxia-inducible factors within the tumor microenvironment or modulating mitochondrial dynamics could improve PDT and PTT efficacy under hypoxic conditions. Additionally, the development of multifunctional nanoparticles that integrate diagnostic and therapeutic capabilities and theranostic platforms could facilitate real-time monitoring of treatment efficacy, enabling personalized, adaptive therapy. The exploration of NIR-II-responsive nanoparticles also holds great promise, as these systems could overcome current limitations in light penetration, allowing for deeper tissue treatment and expanding the range of diseases that can be effectively targeted.

In addition to technological advancements, progress in biocompatibility, biodegradability and large-scale production will be essential for translating phototherapy into widespread clinical use. Innovations in eco-friendly synthesis methods and cost-effective manufacturing processes will play crucial roles in ensuring accessibility and affordability for a global patient population. In conclusion, the convergence of cutting-edge nanotechnology and an in-depth understanding of molecular mechanisms has positioned nanoparticle-based phototherapy at the forefront of modern medicine. Continued research and innovation will be key to unlocking its full therapeutic potential, paving the way for novel, highly effective treatments for complex and hard-to-treat chronic diseases.
